# Synthesis and Characterization of Dendronized Gold Nanoparticles Bearing Charged Peripheral Groups with Antimicrobial Potential

**DOI:** 10.3390/nano12152610

**Published:** 2022-07-29

**Authors:** Gabriel Perli, Diego L. Bertuzzi, Dênio E. P. Souto, Miguel D. Ramos, Carolyne B. Braga, Samile B. Aguiar, Catia Ornelas

**Affiliations:** 1 Institute of Chemistry, University of Campinas, Campinas 13083-970, Brazil; perligabriel@gmail.com (G.P.); diego.bertuzzi@hotmail.com (D.L.B.); denio.souto@ufpr.br (D.E.P.S.); migueljr.qma@gmail.com (M.D.R.); carolbbraga7@gmail.com (C.B.B.); samilede17@gmail.com (S.B.A.); 2 Laboratorio de Espectrometria, Sensores e Biossensores, Departamento de Quimica, Universidade Federal do Paraná (UFPR), Curitiba 81531-980, Brazil

**Keywords:** macromolecules, dendrons, gold nanoparticles, dendronized nanoparticles, antimicrobial, drug delivery

## Abstract

Dendronized gold nanoparticles (AuNPs) were synthesized bearing charged peripheral groups. Two novel AB_3_-type dendrons were synthesized with a thiol group at the focal point followed by their attachment to AuNPs. Dendrons were designed to have nine charged peripheral groups (carboxyl or amine), glycol solubilizing, units and one thiol moiety at the focal point. Both dendrons and all intermediates were synthesized in high yields and characterized by nuclear magnetic resonance spectroscopy (NMR) and mass spectrometry (MS). The amine- and carboxyl-terminated dendrons were used to functionalize gold nanoparticles (AuNPs) previously stabilized with citrate. The nanoparticles’ diameters and their colloidal stability were investigated using dynamic light scattering (DLS). The size and morphology of the dendronized AuNPs were evaluated by scanning electron microscopy (SEM), which revealed individual particles with no aggregation after replacement of citrate by the dendrons, in agreement with the DLS data. The absorption spectroscopy reveals a prominent plasmonic band at 560 nm for all AuNPs. The zeta potential further confirmed the expected charged structures of the dendronized AuNPs. Considering all the physical–chemical properties of the charged dendronized AuNPs developed in this work, these AuNPs might be used as a weapon against multi-drug resistant bacterial infections.

## 1. Introduction

The evolution of multi-drug resistant (MDR) microorganisms has become a severe threat to public health worldwide. According to the last predictions, infections caused by antimicrobial-resistant germs will result in 10 million annual deaths by 2050, exceeding cancer-related mortality [1,2,3]. Due to the risk of these infections and the lack of innovation in this area over the last fifteen years, the quest for new antimicrobial strategies has played a crucial role in science [1,4,5,6]. Nanoparticles (NPs) have been proposed as great candidates for such therapeutic applications owing to their unique characteristics. NPs have a high surface-to-volume ratio that enables enhanced contact with the target organisms [7,8,9,10,11]. NPs can also interact with bacterial cells to regulate the uptake and molecular pathways [12,13,14]. In particular, gold nanoparticles (AuNPs) exhibit additional attributes to help in this endeavor, including chemical stability, biocompatibility, commercial availability, easy functionalization, and high intrinsic antimicrobial activity [15,16,17,18,19]. AuNPs also possess remarkable optical properties related to plasmon absorption, which allow their use in photothermal-induced antibacterial therapy [20,21].

Several reports show enhanced activity of AuNPs bearing antibacterial agents, making clear that the combination of classical antibiotics and AuNPs is a powerful strategy to overcome antibiotic resistance [22,23]. Payne et al. reported that AuNPs grafted with kanamycin have significant antibacterial activity against Gram-positive *Staphylococcus epidermis* and the Gram-negative *Enterobacter aerogenes* [24]. Rattanata et al. observed enhanced bactericidal activity of gallic acid with AuNPs, and demonstrated that AuNPs were able to deliver gallic acid through the bacterial cell membrane and interact with DNA [25].

The intrinsic antimicrobial activity of AuNPs has been shown to be dependent on the nanoparticles’ size, surface chemistry, and type of ligands [8,9,16,26,27,28,29,30]. In this context, the use of dendrimers or dendrons to coat AuNPs is an interesting approach due to the combination of desirable properties from both nanomaterials, since dendrimers and dendrons also exhibit intrinsic antimicrobial activity that is dependent on the dendrons’ functionalization [31,32,33].

Dendrimers and dendrons are a special class of macromolecules with monodispersed architectures and a high density of terminal functional groups [34,35,36]. These molecules are synthesized in a stepwise fashion resulting in well-defined structures [34,35]. The controlled synthetic strategies used to build dendrimers and dendrons allow precise control over the nanoscale size, shape, number, nature, and location of functional groups [37,38].

Dendrimers are commonly radially symmetric macromolecules, whereas dendrons have *AB_x_* branched structures: where *B* represents the terminal functional groups and *A* represents one reactive group at the focal point [34,35,39]. The functional group at the focal point can be used as an anchor for stabilizing metal NPs [40,41], and the active termini may be used to attach a high density of charged groups per macromolecule [42,43,44]. Indeed, their high performance in biomedical applications arise from multivalent interactions and secondary interactions between the dendrimer termini with biomolecules on the target human cells or bacteria [42,45,46,47,48].

For instance, classical antibiotics, charged groups, targeting groups, and/or solubilizing units may be attached to a single dendrimer, allowing the construction of novel smart carriers with tunable properties [49,50,51]. Although the use of branched molecules as nanocarriers is a well-investigated area, there is room for developing dendron-based antimicrobials [42].

Herein, we report the synthesis of two novel well-defined dendrons designed to have amide and glycol solubilizing units in the backbone, one thiol group at the focal point, and nine carboxyl or amine termini. The ethylene glycol derivatives were used to improve water solubility and biocompatibility. The thiol group at the focal point ensures the anchoring on AuNPs, and the charged terminal groups were designed to enhance the antimicrobial effect by interacting with the outer membranes of bacteria.

To decrease the steric hindrance at the focal point of dendrons, a bi-functional glycol spacer was designed to have a protected thiol group and a free carboxylic acid. A second-generation Newkome-type dendron was synthesized and decorated with ethylene glycol derivatives. The resultant dendrons were then attached to the synthesized glycol spacer via amidation. The final dendrons with a free thiol group at the focal point were obtained after sequential deprotection reactions. The dendrons were attached to the surface of AuNPs through the ligand exchange strategy with AuNPs stabilized with citrate. The effect of the ligand replacement over the size, stability, charge, and electronic properties of the dendron-coated AuNPs was analyzed using dynamic light scattering (DLS), zeta potential, UV-vis spectroscopy, and scanning electron microscopy (SEM). We rationalized that the combination of the intrinsic antimicrobial properties of AuNPs and dendrimers allied to the positively or negatively charged surface of the new dendronized AuNPs may result in nanoparticles with enhanced antimicrobial activity.

## 2. Materials and Methods

### 2.1. Materials

All chemicals were purchased from Sigma–Aldrich (St. Louis, MO, USA) and used without further purification. Reactions dealing with air- and moisture-sensitive compounds were carried out using a Schlenk line. Solvents were freshly distilled using a proper drying agent for each case, except for dimethylformamide (DMF), which was purchased as an anhydrous grade in a sealed flask and used under nitrogen atmosphere.

### 2.2. Methods

#### 2.2.1. Synthesis of Compound **2**

In a Schlenk flask, tert-Butyl 12-hydroxy-4,7,10-trioxadodecanoate (0.90 mmol, 250.00 mg), N,N-diisopropylethylamine (1.4 eq, 1.26 mmol, 163.85 mg, 0.220 mL), and p-toluenesulfonyl chloride (1.3 eq, 1.17 mmol, 223.1 mg) were dissolved in 10 mL of anhydrous dichloromethane. The mixture was stirred under N_2_ atmosphere for 12 h at room temperature. The solvent was removed using rotary evaporation and the crude product was purified by column chromatography using silica gel and dichloromethane:methanol (solvent mixture gradient from 100:0 until 95:5) as eluent. Compound **2** was obtained as a colorless oil in 98% of yield (381.1 mg). ^1^H NMR (CDCl_3_, 500 MHz), δ (ppm): 7.75 (d, *J* = 8.0 Hz, Ar*H,*, 2H), 7.30 (d, *J* = 8.0 Hz, Ar*H*, 2H), 4.12 (t, *J* = 5 Hz, C*H*_2_O, 2H), 3.66–3.52 (m, C*H*_2_OC*H*_2_C*H*_2_OC*H*_2_C*H*_2_O, 10H), 2.45 (t, *J* = 6.5 Hz, C*H*_2_CO, 2H), 2.39 (s, ArC*H*_3_, 3H), 1.39 (s, COOC(C*H*_3_)_3_, 9H). ¹³C NMR (CD,Cl_3_, 500 MHz), δ (ppm): 171.9 (*C*OOtBu), 144.8 (Ar*C*_q_), 133.0 (Ar*C*_q_), 129.8 (Ar*C*H), 127.9 (Ar*C*H), 80.5 *C*(CH_3_)_3_, 70.7–66.9 (*C*H_2_O*C*H_2_*C*H_2_O*C*H_2_*C*H_2_O), 36.3 (*C*H_2_COOtBu), 28.1 C(*C*H_3_)_3_, 21.6 (Ar*C*H_3_). ESI-QTOF-MS [M+H]^+^ *m*/*z* calcd for C_20_H_32_O_8_SH: 433.18, found: 433.23.

#### 2.2.2. Synthesis of Compound **3**

In a round-bottom flask, compound **2** (0.81 mmol, 381.1 mg) was dissolved in 1.5 mL of deionized water and 13.5 mL of formic acid. The mixture was stirred at room temperature for 12 h. The aqueous solution was evaporated to dryness using rotary evaporation, and the resulting solid was washed with diethyl ether (3 × 30 mL). Compound **3** was obtained as a white powder in quantitative yield (301.6 mg). ^1^H NMR (CDCl_3_, 500 MHz), δ (ppm): 7.80 (d, *J* = 8.0 Hz, Ar*H*, 2H), 7.32 (d, *J* = 8.0 Hz, Ar*H*, 2H), 4.16 (t, *J* = 5 Hz, C*H*_2_O, 2H), 3.79–3.60 (m, *C*H_2_O*C*H_2_*C*H_2_O*C*H_2_*C*H_2_O, 10H), 2.67 (t, *J* = 6.5 Hz, C*H*_2_CO, 2H), 2.42 (s, ArC*H*_3_, 3H). ¹³C NMR (CDCl_3_, 500 MHz), δ (ppm): 176.2 (*C*OOH), 145.1 (Ar*C*_q_), 131.5 (Ar*C*_q_), 130.0 (Ar*C*H), 128.1 (Ar*C*H), 80.5 *C*(CH_3_)_3_, 70.8–66.4 (*C*H_2_O*C*H_2_*C*H_2_O*C*H_2_*C*H_2_O), 34.8 (*C*H_2_COOH), 21.8 (Ar*C*H_3_). ESI-QTOF-MS [M+H]^+^ *m*/*z* calcd for C_16_H_24_O_8_SH: 377.12, found: 377.21.

#### 2.2.3. Synthesis of Compound **4**

In an air-free Schlenk flask, compound **3** (0.80 mmol, 301.6 mg) and potassium thioacetate (1.5 eq, 1.2 mmol, 137.1 mg) were dissolved in 10 mL of anhydrous N,N-dimethylformamide. The mixture was stirred under N_2_ atmosphere for 24 h at room temperature. The reaction mixture was washed with deionized water (3 × 10 mL), and the organic phase was dried over Na_2_SO_4_, filtered, and concentrated under vacuum. The crude product was further purified by column chromatography using silica gel and dichloromethane: methanol (solvent mixture gradient from 100:0 to 90:10) as eluent. Compound **4** was obtained as a yellowish oil in 85% of yield (190.5 mg). ^1^H NMR (CDCl_3_, 500 MHz), δ (ppm): 3.78–3.58 (m, C*H*_2_OC*H*_2_C*H*_2_OC*H*_2_C*H*_2_O, 10H), 3.09 (t, *J* = 8.5 Hz, SC*H*_2_O, 2H), 2.64 (t, *J* = 7.5 Hz, C*H*_2_COOH), 2.33 (s, S(CO)C*H*_3_, 3H). ¹³C NMR (CDCl_3_, 500 MHz), δ (ppm): 195.9 (CH_3_(*C*O)S) 176.2 (*C*OOH), 70.7–66.5 (*C*H_2_O*C*H_2_*C*H_2_O*C*H_2_*C*H_2_O), 34.9 (*C*H_2_S(CO)CH_3_), 30.7 (*C*H_2_COOH), 29.0 (S(CO)*C*H_3_). ESI-QTOF-MS [M+H]^+^ *m*/*z* calcd for C_11_H_20_O_6_SH: 281.10, found: 281.11.

#### 2.2.4. Synthesis of Compound **6**

In an air-free Schlenk flask, compound **5** (0.51 mmol, 590 mg), N,N-diisopropylethylamine (1.4 eq, 0.71 mmol, 92.3 mg, 0.124 mL), and HATU (1.4 eq, 0.71 mmol, 0.271 g) were dissolved in 15 mL of anhydrous N,N-dimethylformamide. The mixture was stirred for 15 min under nitrogen atmosphere, and the N-Boc-2,2′-(ethylenedioxy)diethylamine (9.5 eq, 4.85 mmol, 1.2 g, 1.15 mL) was slowly added using a proper needle and syringe. The mixture was stirred for 48 h at room temperature and under nitrogen atmosphere. The organic solvent was removed by rotary evaporation, followed by washing with deionized water (10 × 20 mL). The product was further purified by column chromatography using silica gel and dichloromethane:methanol (solvent mixture gradient from 100:0 to 95:5) as eluent. Compound **6** was obtained as a yellowish oil with 85% of yield (1.4 g). ^1^H NMR (CDCl_3_, 600 MHz), δ (ppm): 7.74 (d, *J* = 8.7 Hz, Ar*H*, 2H), 7.63 (d, *J* = 8.7 Hz, Ar*H*, 2H), 7.37 (t, *J* = 8.4 Hz, Ar*H*, 2H), 7.30 (t, *J* = 8.4 Hz, Ar*H*, 2H), 7.08 (s, CON*H*, 9H), 6.19 (s, CON*H*, 1H), 5.35 (s, CH_2_CON*H*, 9H), 4.26 (br d, C*H*_2_CONH, 2H), 4.18 (br t, C*H*CH_2_CONH, 1H), 3.56–3.51 (br t, C*H*_2_OC*H*_2_C*H*_2_OC*H*_2_, 72H), 3.50–3.48 (br t, CONHC*H*_2_, 18H), 3.36–3.27 (br t, C*H*_2_NHCOOtBu, 18H), 2.18 (br t, C*H*_2_CO, 24H), 1.98 (br t, HCC*H*_2_, 24H), 1.41 (s, (CO)OC(C*H*_3_)_3_), 81H). ¹³C NMR (CDCl_3_, 600 MHz), δ (ppm): 173.6 (CH_2_(*C*O)NH), 173.4 (CH_2_(*C*O)NH), 156.3 (NH(*C*O)OtBu), 155.0 (NH(*C*O)OtBu), 144.7–120.1 (Fmoc-Ar 13C), 79.3 (*C*_q_(CH_3_)_3_) 70.3–69.8 (*C*H_2_O*C*H_2_*C*H_2_O), 66.2 (C*C*H_2_O), 58.0 (O*C*H_2_CH_2_NH), 57.4 (NH*C*_q_), 47.4 (*C*CH_2_O), 41.7 (NH*C*_q_), 40.4 (HN*C*H_2_), 39.4 (*C*H_2_NH), 31.6 (*C*H_2_CONH), 30.9 (HNC_q_*C*H_2_), 29.8–28.6 (*C*H_2_*C*H_2_CO), 28.6 (OC(*C*H_3_)_3_). MALDI-TOF-MS [M+Na]^+^ *m*/*z* calcd for C_154_H_270_N_22_O_50_Na: 3251.35, found: 3251.70.

#### 2.2.5. Synthesis of Compound **7**

In an air-free Schlenk flask, compound **5** (0.51 mmol, 590 mg), N,N-diisopropylethylamine (1.4 eq, 0.71 mmol, 92.3 mg, 0.124 mL), and HATU (1.4 eq, 0.71 mmol, 0.271 g) were dissolved in 15 mL of anhydrous N,N-dimethylformamide. The mixture was stirred for 15 min under nitrogen atmosphere, and the tert-butyl 12-amino-4,7,10-trioxadodecanoate (9.5 eq, 4.85 mmol, 1.34 g, 1.20 mL) was slowly added using a proper needle and syringe. The mixture was stirred for 48 h at room temperature and under nitrogen atmosphere. The organic solvent was removed by rotary evaporation, followed by washing with deionized water (10 × 20 mL). The product was further purified by column chromatography using silica gel and dichloromethane:methanol (solvent mixture gradient from 100:0 to 94:6) as eluent. Compound **7** was obtained as a yellow oil with 95% of yield (1.69 g). ^1^H NMR (CDCl_3_, 600 MHz), δ (ppm): 8.4 (d, *J* = 8.7 Hz, Ar*H*, 2H), 7.63 (br d, Ar*H*, 2H), 7.35 (t, *J* = 8.4 Hz, Ar*H*, 2H), 7.27 (t, *J* = 8.4 Hz, Ar*H*, 2H), 4.47 (br d, C*H*_2_CONH, 2H), 4.17 (br t, C*H*CH_2_CONH, 1H), 3.68 (t, *J* = 7.8 Hz, OC*H*_2_C*H*_2_, 18H), 3.67–3.65 (m, OC*H*_2_C*H*_2_OC*H*_2_C*H*_2_O, 72H), 3.47 (br t, C*H*_2_O, 18H), 3.33 (br t, NHC*H*_2_, 18H), 2.48 (t, *J* = 7.8 Hz, C*H*_2_(CO)OtBu, 18H), 2.47 (br t, C*H*_2_CO, 24H), 2.18 (br t, HCC*H*_2_, 24H), 1.41 (s, (CO)OC(C*H*_3_)_3_), 81H). ¹³C NMR (CDCl_3_, 600 MHz), δ (ppm): 173.7 ((*C*O)OtBu), 171.2 (CH_2_(*C*O)NH), 155.1 (NH(*C*O)C_q_), 144.2–120.0 (Fmoc-Ar 13C), 80.7 (*C*_q_(CH_3_)_3_), 66.3–57.4 (NH*C*_q_), 47.3 (C*C*H_2_O), 39.4 (HN*C*H_2_), 36.3 (*C*H_2_(CO)OtBu), 31.1–29.8 (HNC_q_*C*H_2_*C*H_2_CONH), 30.9 (HNC_q_*C*H_2_), 28.2 (OC(*C*H_3_)_3_). MALDI-TOF-MS [M+H_2_O]^+^ *m*/*z* calcd for C_172_H_297_N_13_O_59_H_2_O: 3509.06, found: 3509.57.

#### 2.2.6. Synthesis of Compound **8**

In a Schlenk flask, compound **6** (0.4 mmol, 1.4 g) was dissolved in 30 mL of N,N-dimethylformamide, and N-methyl-morpholine (1.2 eq, 0.5 mmol, 41.8 mg, 46 μL) was added. The mixture was stirred for 4 h. The excess of N,N-dimethylformamide was removed by rotary evaporation, followed by washing with deionized water (10 × 20 mL). The product was further purified by column chromatography using silica gel and dichloromethane: methanol (solvent mixture gradient from 100:0 to 94:6) as eluent. The compound **8** was obtained as a yellow oil in quantitative yield (1.2 g). ^1^H NMR (CDCl_3_, 600 MHz), δ (ppm): 7.44 (s, CON*H*, 9H), 5.371 (s, CON*H*, 9H), 3.93 (s, N*H*_2_), 3,57 (br t, OC*H*_2_C*H*_2_O, 36H), 3.51 (br t, HNC*H*_2_C*H*_2_O, 36H), 3.36 (br t, OC*H*_2_CH_2_NH, 18H), 3.26 (br t, OCH_2_C*H*_2_NH, 18H), 2.44 (br t, C*H*_2_(CO)NH, 6H), 2.18 (br t, C*H*_2_(CO)NH, 18H), 1.96 (br t, NHCH_2_C*H*_2_(CO)NH, 18H), 1.40 (s, (CO)OC(C*H*_3_)_3_), 81H). ¹³C NMR (CDCl_3_, 600 MHz), δ (ppm): 173.9 (CH_2_(*C*O)NH), 173.4 (CH_2_(*C*O)NH), 156.3 (NH(*C*O)OtBu), 79.3 (O*C*(CH_3_)_3_), 70.3 (O*C*H_2_*C*H_2_O), 70.2 (CH_2_*C*H_2_O), 64.1 (*C*H_2_O), 43.8 (OOCNH*C*_q_), 40.4 (NH*C*H_2_CH_2_O), 39.4 (*C*H_2_NH), 30.7 (*C*H_2_CONH), 29.8 (HNC_q_*C*H_2_), 28.5 (OC(*C*H_3_)_3_). MALDI-TOF-MS [M+Na]^+^ *m*/*z* calcd for C_139_H_260_N_22_O_48_H: 3006.86, found: 3007.80.

#### 2.2.7. Synthesis of Compound **9**

In a Schlenk flask, compound **7** (0.48 mmol, 1.69 g) was dissolved in 30 mL of N,N-dimethylformamide, and N-methyl-morpholine (1.2 eq, 0.58 mmol, 50.6 mg, 50 μL) was added. The mixture was stirred for 4 h. The excess of N,N-dimethylformamide was removed by rotary evaporation, followed by washing with deionized water (10 × 20 mL). The product was further purified by column chromatography using silica gel and dichloromethane: methanol (solvent mixture gradient from 100:0 to 94:6) as eluent. The compound **9** was obtained as a yellow oil in quantitative yield (1.57 g). ^1^H NMR (CDCl_3_, 600 MHz), δ (ppm): 7.26 (s, CON*H*, 9H), 3.70 (t, *J* = 6 Hz, OC*H*_2_C*H*_2_, 18H), 3.68 (t, *J* = 7.8 Hz, OC*H*_2_C*H*_2_, 18H), 3.61 (m, OC*H*_2_C*H*_2_OC*H*_2_C*H*_2_O, 72H), 3.52 (br t, C*H*_2_O, 18H), 3.37 (br t, NHC*H*_2_, 18H), 2.50 (t, *J* = 7.8 Hz, C*H*_2_(CO)OtBu, 18H), 2.18 (br t, C*H*_2_CO, 24H), 1.97 (br t, HCC*H*_2_, 24H), 1,68 (br t, H_2_NC_q_C*H*_2_, 6H), 1.41 (s, (CO)OC(C*H*_3_)_3_), 81H). ¹³C NMR (CDCl_3_, 600 MHz), δ (ppm): 173.7 (NH(*C*O)OtBu), 171.1 (CH_2_(*C*O)NH), 80.7 (O*C*(CH_3_)_3_), 70.6–66.9 (*C*H_2_O*C*H_2_*C*H_2_O*C*H_2_*C*H_2_O*C*H_2_), 58.1 (NH_2_*C*_q_), 39.5 (*C*H_2_NH), 36.4 (*C*H_2_CONH), 30.8 (HNC_q_*C*H_2_), 28.54(OC(*C*H_3_)_3_). MALDI-TOF-MS [M+Na]^+^ *m*/*z* calcd for C_157_H_287_N_13_O_57_H: 3267.99, found: 3269.47.

#### 2.2.8. Synthesis of Compound **10**

In an air-free Schlenk flask, compound **4** (1.4 eq, 0.56 mmol, 156.8 mg), N,N-diisopropylethylamine (1.4 eq, 0.56 mmol, 72.4 mg, 0.094 mL), and HATU (1.4 eq, 0.56 mmol, 212.9 mg) were dissolved in 20 mL of anhydrous N,N-dimethylformamide. The mixture was stirred for 15 min, and compound **8** (0.4 mmol, 1.2 g) was added. The mixture was stirred for 24 h at room temperature under nitrogen atmosphere. The solvent was removed by rotary evaporation and by washing with deionized water (10 × 20 mL). The product was further purified by column chromatography using silica gel and dichloromethane: methanol (solvent mixture gradient from 100:0 to 90:10) as eluent. Compound **10** was obtained as a yellow oil in 95% of yield (1.24 g). ^1^H NMR (CDCl_3_, 600 MHz), δ (ppm): 7.57 (s, CON*H*, 9H), 3.60 (br t, OC*H*_2_C*H*_2_O, 36H), 3.51 (br t, HNCH_2_C*H*_2_O, 18H), 3.39 (br t, OCH_2_C*H*_2_NH, 18H), 3.30 (br t, SC*H*_2_CH_2_, 2H), 2.54–2.41 (br t, OC*H*_2_C*H*_2_OC*H*_2_C*H*_2_OCH_2_,10H), 2.33–2.18 C*H*_2_(CO)NH, 2.33 (S(CO)C*H*_3_, 3H), 2.27 (br t, C*H*_2_(CO)NH, 24H), 2.18 (br t, C*H*_2_(CO)NH, 18H), 1.42 (s, (CO)OC(C*H*_3_)_3_), 81H). ¹³C NMR (CDCl_3_, 600 MHz), δ (ppm): 195.3 (CH_3_(*C*O)S), 173.9 (CH_2_(*C*O)NH), 156.3 (NH(*C*O)OtBu), 77.3 (O*C*(CH_3_)_3_), 70.3 (O*C*H_2_*C*H_2_O), 70.2 (*C*H_2_O*C*H_2_*C*H_2_O*C*H_2_*C*H_2_OCH_2_), 64.1 (*C*H_2_O), 40.3 (OOCNH*C*_q_), 39.4 (NHCH_2_*C*H_2_O), 39.4 (*C*H_2_NH), 30.7 (*C*H_2_CONH), 29.8 (HNC_q_*C*H_2_), 28.5 (OC(*C*H_3_)_3_). MALDI-TOF-MS [M+Na]^+^ *m*/*z* calcd for C_150_H_278_N_22_O_53_Na: 3291.9, found: 3291.6.

#### 2.2.9. Synthesis of Compound **11**

In an air-free Schlenk flask, compound **4** (1.4 eq, 0.67 mmol, 156.8 mg), N,N-diisopropylethylamine (1.4 eq, 0.67 mmol, 86.9 mg, 0.117 mL), and HATU (1.4 eq, 0.67 mmol, 254.7 mg) were dissolved in 20 mL of anhydrous N,N-dimethylformamide. The mixture was stirred for 15 min, and compound **9** (0.48 mmol, 1.57 g) was added. The mixture was stirred for 24 h at room temperature under nitrogen atmosphere. The solvent was removed by rotary evaporation and washed with deionized water (10 × 20 mL). The product was further purified by column chromatography using silica gel and dichloromethane: methanol (solvent mixture gradient from 100:0 to 90:10) as eluent. Compound **11** was obtained as a yellow oil in 87% of yield (1.47 g). ^1^H NMR (CDCl_3_, 600 MHz), δ (ppm): 7.26 (s, CON*H*, 9H), 3.68 (br t, OC*H*_2_C*H*_2_, 18H), 3.69–3.61 (m, OC*H*_2_C*H*_2_OC*H*_2_CH_2_O, 72H), 3.68 (t, *J* = 7.8 Hz, OC*H*_2_C*H*_2_, 18H), 3.52 (br t, C*H*_2_O, 18H), 3.37 (br t, NHC*H*_2_, 18H), 2.50 (t, *J* = 7.8 Hz, C*H*_2_(CO)OtBu, 18H), 2.28 (br t, C*H*_2_(CO)NH, 2H), 2.31 (s, *H*_3_C(CO)S, 3H), 2.16 (br t, C*H*_2_CO, 24H), 1.97 (br t, HNC*H*_2_, 24H), 1.95 (br t, NHC*H*_2_, 6H), 1.41 (s, (CO)OC(C*H*_3_)_3_), 81H). ¹³C NMR (CDCl_3_, 600 MHz), δ (ppm): 195.9 (H_3_C(*C*O)S), 173.8 ((*C*O)OtBu), 171.1 (CH_2_(*C*O)NH), 80.7 (O*C*(CH_3_)_3_), 70.6–66.9 (*C*H_2_O*C*H_2_*C*H_2_O*C*H_2_*C*H_2_OCH_2_), 58.1 (NH_2_*C*_q_), 39.5 (*C*H_2_NH), 36.4 (*C*H_2_CONH), 30.8 (HNC_q_*C*H_2_), 28.5 (OC(*C*H_3_)_3_).

#### 2.2.10. Synthesis of Compound **12**

In a round-bottom flask, compound **10** (0.38 mmol, 1.24 g) was dissolved in 20 mL of deionized water and 80 mL of formic acid. The reaction mixture was stirred for 48 h. The aqueous solution was removed using rotary evaporation. The solid product was solubilized in dichloromethane and washed ten times with a cold saturated sodium carbonate solution. The organic solution was dried with Na_2_SO_4_, filtered, and the dichloromethane was evaporated yielding the compound **12** as a yellow oil. ^1^H NMR (D_2_O, 600 MHz), δ (ppm): 9.63, 8.25, and 7.98 (br s, N*H,* and S*H*), 3.49 (br s, OC*H*_2_C*H*_2_), 3.25 (br s, NHC*H*_2_), 3,08 (br s, C*H*_2_(CO)NH), 2.08 (br t, C*H*_2_CO), 1.82 (br t, HNC*H*_2_). ¹³C NMR (D_2_O, 600 MHz), δ (ppm): 175.8 (CH_2_(*C*O)NH), 69.5–66.4 (*C*H_2_O*C*H_2_*C*H_2_O*C*H_2_*C*H_2_OCH_2_), 57.9 (NH_2_*C*_q_), 39.0–38.0 (*C*H_2_NH), 37.8 (*C*H_2_CONH), 30.5 (HNC_q_*C*H_2_). MALDI-TOF-MS [M+H]^+^ *m*/*z* calcd for C_103_H_205_N_22_O_34_S: 2326.40, found: 2428.02.

#### 2.2.11. Synthesis of Compound **13**

In a round bottom flask, compound **11** (0.38 mmol, 1.47 g) was dissolved in 20 mL of deionized water and 80 mL of formic acid. The reaction mixture was stirred for 48 h. The aqueous solution was removed using rotary evaporation. The solid product was solubilized in dichloromethane and washed ten times with a cold saturated sodium carbonate solution. The organic solution was dried with Na_2_SO_4_, filtered, and the dichloromethane was evaporated yielding the compound **13** as a yellow oil. ^1^H NMR (D_2_O, 600 MHz), δ (ppm): 7.41 (br s, N*H,* and S*H*), 3.74–3.55 (br s, OC*H*_2_C*H*_2_), 2.54 (br s, C*H*_2_(CO)NH), 2.22 (br t, C*H*_2_CO), 2.00 (br t, HNC*H*_2_). ¹³C NMR (D_2_O, 600 MHz), δ (ppm): 175.8 (CH_2_(*C*O)NH), 69.5–66.4 (*C*H_2_O*C*H_2_*C*H_2_O*C*H_2_*C*H_2_OCH_2_), 57.9 (NH_2_*C*_q_), 39.1–38.9 (*C*H_2_NH and *C*H_2_CONH) 29.9 (HNC_q_*C*H_2_). MALDI-TOF-MS [M+H]^+^ *m*/*z* calcd for C_130_H_231_N_13_O_61_S: 2982.5 found: 2984.1.

### 2.3. Nuclear Magnetic Resonance (NMR)

One-dimensional NMR spectra of ^1^H and ^13^C were obtained on either a Bruker Avance-III HD 250 MHz, Bruker AVANCE 400, Bruker AVANCE 500, or a Bruker Avance-III 600 MHz spectrometer. The spectra were obtained at 25 °C, and deuterated solvents were used as the lock. Chemical shifts (δ) are reported in parts per million (ppm) using the deuterated solvent residual peaks as the reference.

### 2.4. Mass Spectrometry (MS)

High-resolution mass spectra were acquired in a Waters Xevo Q-TOF (ESI-QTOF) mass spectrometer. Samples solution in methanol or acetonitrile were injected into the mass spectrometer. Before each analysis, the spectrometer was externally calibrated with phosphoric acid ranging from 98 to 1300 *m*/*z*. MALDI-TOF mass spectra were recorded on a Bruker Autoflex III MALDI-TOF MS or on a Bruker Microflex II MALDI-TOF MS. Samples were prepared by the dried droplet method, using the universal MALDI-TOF matrix (1:1 mixture of 2,5-dihydroxybenzoic acid and α-cyano-4-hydroxycinnamic acid). A fresh matrix solution was prepared for each experiment at the concentration of 10 mg/mL in acetonitrile and water (1:1, *v*/*v*) containing 2% of formic acid.

### 2.5. General Procedure for Functionalization of Gold Nanoparticles

The citrated-stabilized gold nanoparticles with spherical shape and mean diameter of 60 nm were purchased from Sigma–Aldrich as a solution in citrate buffered medium. Both dendrons were mixed with the gold nanoparticles in a molar ratio of 5 dendrons per 1 AuNP in deionized water, for 48 h at room temperature and under gentle stirring, in the dark. The dendronized gold nanoparticles were purified by dialysis using benzoylated dialysis tubing with 3 kDa molecular weight cut-off (MWCO) to remove the citrate molecules. Approximately 10 cm of the dialysis membrane was washed with deionized water and subsequently loaded with the samples of gold-dendron conjugates. The bags were closed with dialysis tubing clips and placed inside a beaker containing 1 L of deionized water. The outside water was replaced daily for 72 h. After completing the dialysis process, the samples were lyophilized overnight.

### 2.6. Dynamic Light Scattering (DLS)

The nanoparticles’ hydrodynamic diameter and their polydispersity indices (PDIs) were obtained by dynamic light scattering (DLS). Measurements were carried out at 25 °C on a Zetasizer Nano–ZS ZEN3600 instrument (Malvern Instruments) equipped with a 4 mW He–Ne laser with light wavelength of 632.8 nm, and backscattering angle of 173°, using a disposable DTS 1070 cell. The dendronized gold nanoparticles were redispersed by vortexing in deionized water at a concentration of 1 mg/mL. Each sample was analyzed in triplicate.

### 2.7. Zeta Potential

The zeta potential results are based on the measurement of the velocity of nanoparticles electrophoretic mobility using the laser Doppler anemometry technique. All samples were diluted in ultrapure water at final concentration of 1 mg/mL and the measurements were performed in triplicate at 25 °C using a Zetasizer Nano ZS (Malvern Instruments, Worcester, UK) in a DTS1070 disposable cuvette.

### 2.8. Absorption Spectroscopy in the UV-Vis

The plasmon surface resonance of the citrate- and dendron-stabilized AuNPs were assessed using an UV–vis spectrometer (Agilent HP 8453, Santa Clara, CA, USA). The lyophilized samples were redispersed using the vortex agitation for 10 min, without further energy to disperse the particles and the spectra were recorded in a 1 cm cell.

### 2.9. Scanning Electron Microscopy (SEM)

The surface morphology of both dendronized AuNPs was examined using a Quanta 250 field emission scanning electron microscope (FESEM) (FEI Ltd., Natural Bridge Station, VA, USA) operating at an accelerating voltage of 15 kV. Particle samples were prepared by dropping 10 μL of a nanoparticle dispersion at concentration of 1 mg/mL onto a polished silicon wafer. Samples were allowed to dry overnight in the air. The sputters were not coated with a layer of metallic alloy since AuNPs are good electron conducting materials. Under this condition it is possible to visualize the contrast between the organic (dendrons) and metallic portion (AuNPs).

## 3. Results

**Figure 1:**
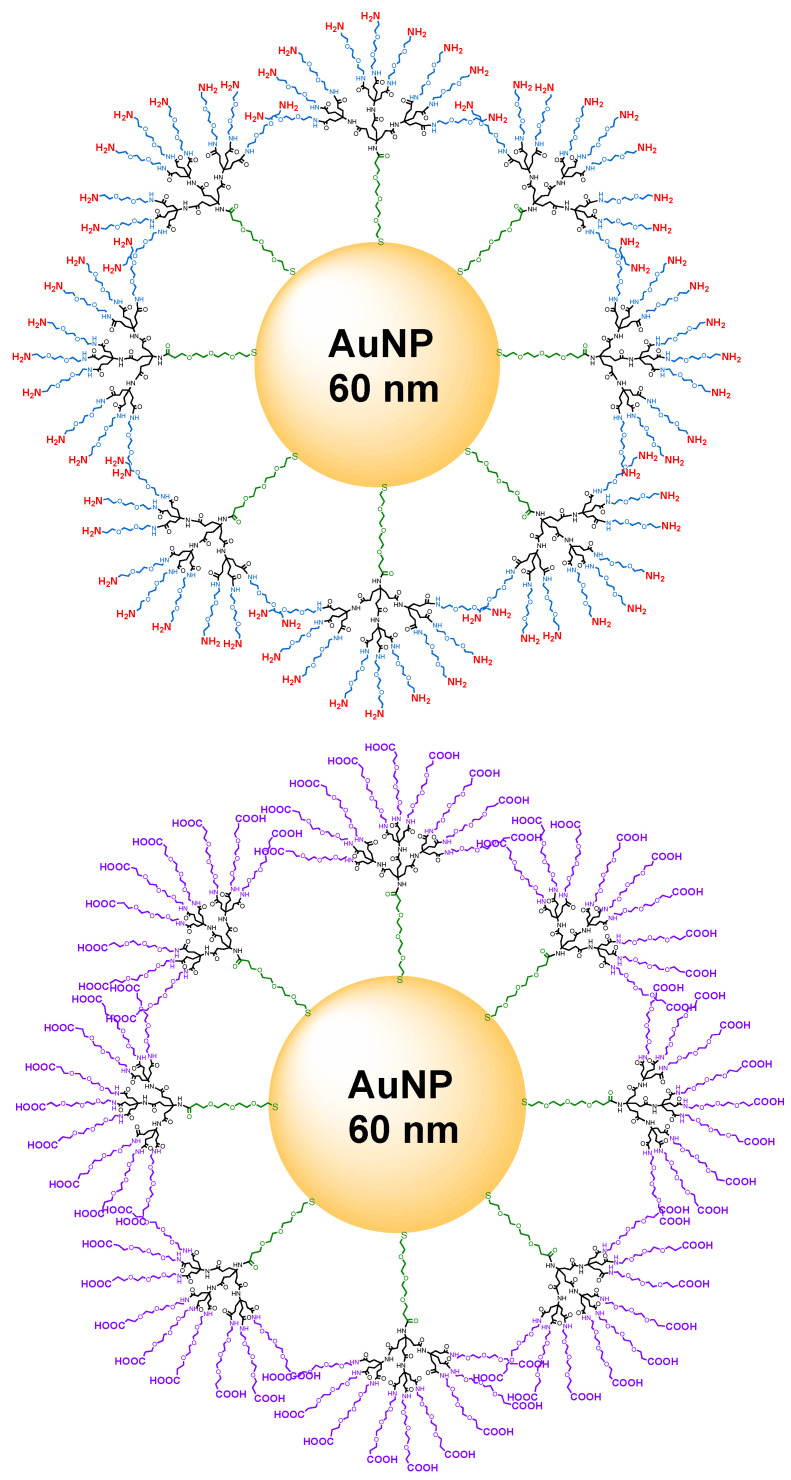
Dendronized gold nanoparticles with amine or carboxylic acid termini synthesized in this work.

In order to obtain NPs with high antimicrobial potential, here we have combined AuNPs with dendrons since both nanostructures possess intrinsic antimicrobial properties. Moreover, the terminal groups on the dendronized AuNP were designed to be negatively or positively charged, which might enhance the antimicrobial activity of these NPs (Figure 1).

Two dendrons were synthesized bearing terminal charged groups, and one thiol moiety at the focal point was responsible for anchoring the dendrons onto the AuNP surface. The amine- and carboxyl-derivative dendrons were designed with polyamide backbones comprising ethylene glycol branches to afford high biodegradability, biocompatibility, and water solubility. Using the AuNPs as a rigid core, it was expected that the peripheral charged groups would remain symmetrically distributed around the AuNPs, enabling an effective interaction between terminal charged groups and the outer membranes of bacteria.

**Scheme 1:**
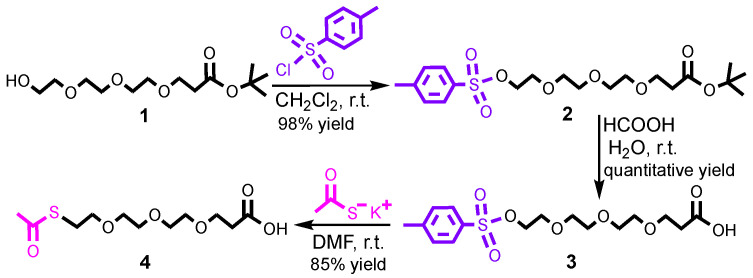
Synthesis of the glycol spacer 4 with a thiol anchoring moiety and a carboxylic acid group.

Herein, a straightforward synthetic route with a set of efficient reactions was essential to complete the synthesis of the two dendrons. The total synthesis of both dendrons was carried out in seven steps. The spacer **4** bearing a thiol end-group and a carboxylic acid group was synthesized in high global yield. The alcohol group on compound **1** was tosylated to generate compound **2** in 98% yield. The carboxylic acid was deprotected using formic acid, affording compound **3** in quantitative yield. Finally, the tosyl group was substituted with thioacetate to provide the target product **4** in 85% yield (Scheme 1).

The ^1^H NMR spectrum of compound **4** exhibits a sharp singlet integrating for three protons at 2.33 ppm corresponding to a methyl group adjacent to the carbonyl of thioester. The ^13^C NMR spectrum of compound **4** shows a signal at 195.9 ppm that is the characteristic shift for thioester carbonyls (see Supplementary Information). The mass spectrum of spacer 4 shows the molecular ion peak (M+Na)^+^ at 303.23 *m*/*z* (calcd for C_11_H_20_O_6_SNa: 303.09 *m*/*z*) further validating the efficacy of the proposed synthetic route.

**Scheme 2:**
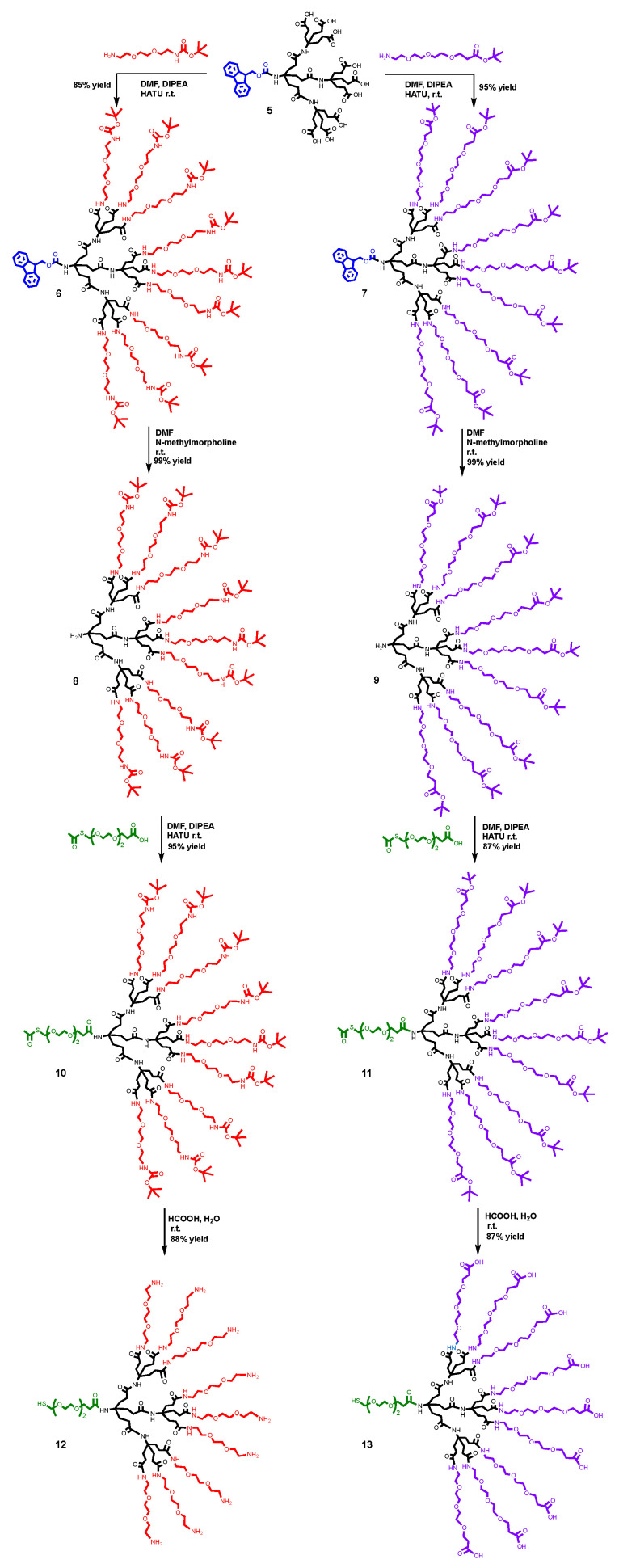
Synthesis of two dendrons with a thiol group at the focal point and nine amine or carboxylic acid termini.

The synthesis of the dendrons’ backbone was carried out according to a previously published work [52]. Newkome-type backbone with 1 → 3 connectivity was built and subsequently functionalized with an amine- or acid carboxylic-glycol derivative (Scheme 2).

The coupling of N-Boc-2,2′-(ethylenedioxy)diethylamine or tert-Butyl-12-amino-4,7,10-trioxadodecanoate with dendron five was achieved using HATU, and dimethylformamide (DMF) as solvent in the presence of N,N-diisopropylethylamine (DIPEA). The products were purified by column chromatography using dichloromethane:methanol as eluent, yielding compounds **6** and **7** as colorless oils. The Fmoc protecting group was removed using the non-nucleophilic base N-methylmorpholine (Scheme 2). The structures of dendrons **6** and **7** were confirmed by NMR spectroscopy and mass spectrometry. In addition to the characteristic peaks of the polyamide scaffold, it is possible to observe signals corresponding to tert-butyloxycarbonyl protons. The signals at approximately 3.50 ppm can be attributed to the protons of the ethylene glycol group (see Supplementary Information).

Spacer **4** was coupled to both dendrons using HATU and DIPEA in DMF. The tert-butyloxycarbonyl groups of the dendrons’ termini were removed using an acidic solution prepared with formic acid. Following the deprotection reaction, the product was dissolved in dichloromethane and washed three times with a saturated sodium carbonate solution. Interestingly, this washing procedure was enough to remove the acetyl group leaving the thiol group unprotected (Scheme 2). Final dendrons **12** and **13** were purified by column chromatography using dichloromethane and methanol, yielding the products as yellowish oils. The target dendrons were characterized by NMR spectroscopy mass spectrometry.

The commercial AuNPs with 60 nm stabilized by citrate were functionalized with the amine- and carboxyl-terminated dendrons through the Au-S bonds. To replace the citrate molecules, both dendrons were stirred in deionized water with the citrate-stabilized AuNPs for 48 h at room temperature, in the dark, in a molar ratio of five dendrons per each AuNP. The AuNPs interact with the amino and carboxyl groups present in the dendron’s structures, however considering the higher affinity of the sulfur–gold bond, the functionalization occurs preferably by the thiol group [53,54,55,56].

**Figure 2:**
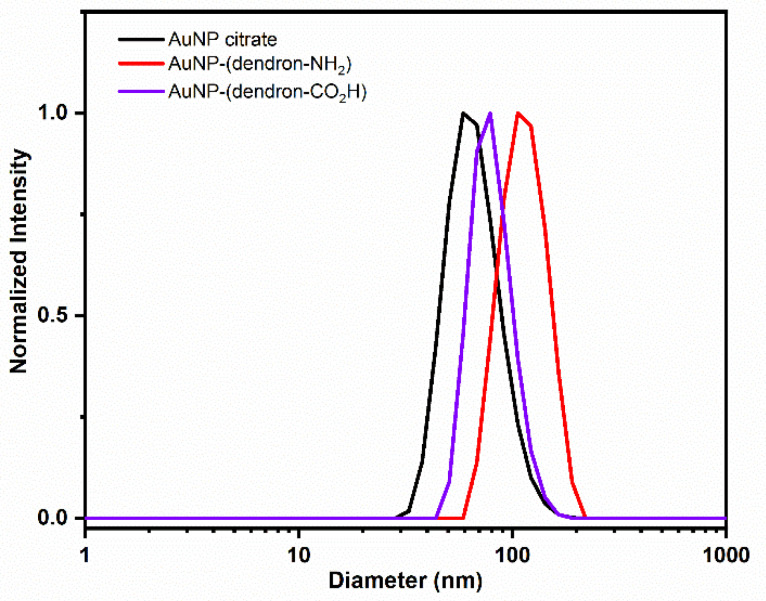
Size distribution profile and the average hydrodynamic diameters obtained by DLS of citrate stabilized AuNPs, cationic dendrimer stabilized AuNPs (AuNP-(dendron-NH_2_)), and anionic dendrimer stabilized AuNP (AuNP-(dendron-CO_2_H)).

Considering that the exchange of the citrate by dendrons may lead to particle coalescence, the colloidal behavior of the dendronized nanoparticles was assessed by dynamic light scattering (DLS) (Figure 2). The size distribution exhibited a shift to larger diameters upon ligand exchange, indicating partial substitution of the citrate molecules by the dendrons. The hydrodynamic diameter of AuNP–citrate is 60 nm, whereas the hydrodynamic diameter of AuNP–(dendron-NH_2_) and AuNP–(dendron-CO_2_H) is 109 nm and 80 nm, respectively (Table 1). Indeed, this increase in the hydrodynamic diameter was expected because the dendrons are larger than the citrate molecules. Moreover, due to the ethylene glycol units, the dendronized AuNPs are highly hydrophilic, consequently, the water molecules will strongly interact with the dendrons’ branches resulting in a water layer around the dendronized AuNPs. This solvation phenomenon justifies the increase in the hydrodynamic diameter. Remarkably, despite the presence of free amine or carboxylic acid groups, the size distributions exhibited monomodal profiles with narrow distributions, suggesting that the nanoparticles present low polydispersities and no aggregation.

Zeta potential measurements were employed to investigate the surface charge variations upon conjugation of the dendrons to the AuNPs. Briefly, zeta potential corresponds to the electric potential in the interfacial double layer at the slipping plane. In other words, zeta potential is the potential difference between the bulk environment and the stationary shell that surrounds the particle. Zeta potential can provide insights into the superficial charge density of AuNPs [57,58,59].

The citrate-stabilized AuNPs presented −49 ± 9.1 mV for zeta potential, and when functionalized with the amine-derived dendron this value increased to +27 ± 6 mV, corroborating the proper substitution of citrate molecules. The positive potential zeta was indeed expected since anchoring the thiol group at the metallic surface exposes the positively charged amine groups around the AuNPs.

The zeta potential analysis also evidenced the replacement of citrate molecules by the acid carboxylic-derivative dendron. The zeta potential value shifted from −49 ± 9 mV to −31 ± 8 mV, which is directly associated with a decrease in the particles’ electrophoretic mobility [58,60,61]. Considering the negligible changes in the continuous phase, the reduction in the electrophoretic mobility is ascribed to the increment of the hydrodynamic diameter and consequent decrease in the hydrodynamic mobility. The decrease in electrophoretic mobility occurs because larger particles pose a greater retardation force [61]. Moreover, the replacement of citrate molecules by branched macromolecules increases the friction coefficient that acts contrary to electrophoretic mobility.

**Figure 3:**
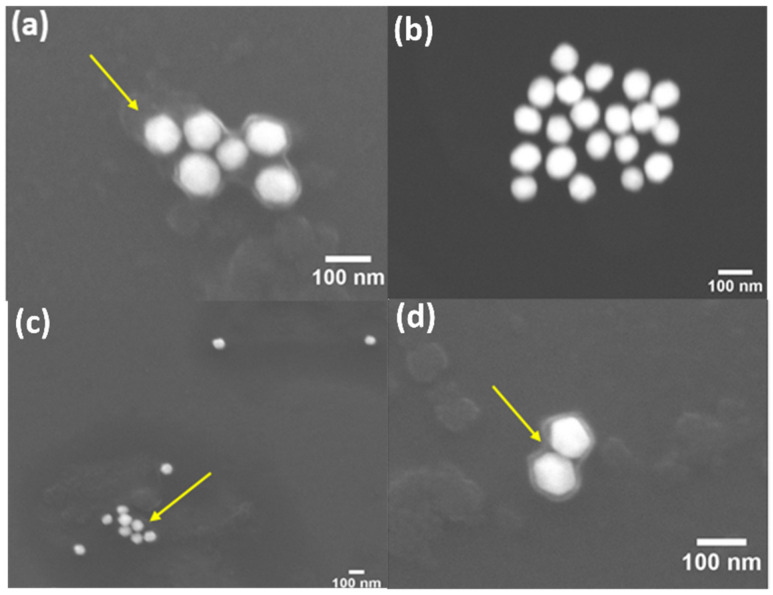
SEM images of AuNPs stabilized by (**a**,**b**) cationic dendronized AuNP-(dendron-NH_2_), and (**c**,**d**) anionic dendronized AuNP-(dendron-CO_2_H). The arrows highlight the contrast between the organic stabilizing and the metallic portion.

The morphology of the dendronized AuNPs was investigated by scanning electron microscopy (SEM), which further confirmed the DLS data. The sputters were not coated with a layer of metallic alloy since AuNPs are good electron conducting materials. Under this condition it was possible to visualize the contrast between the organic and metallic portion, that corresponds to the dendron stabilizers and the AuNPs, respectively (Figure 3). The SEM analysis revealed no signal of aggregation phenomena, showing individual AuNPs with a surrounding layer of organic material (dendrons).

**Figure 4:**
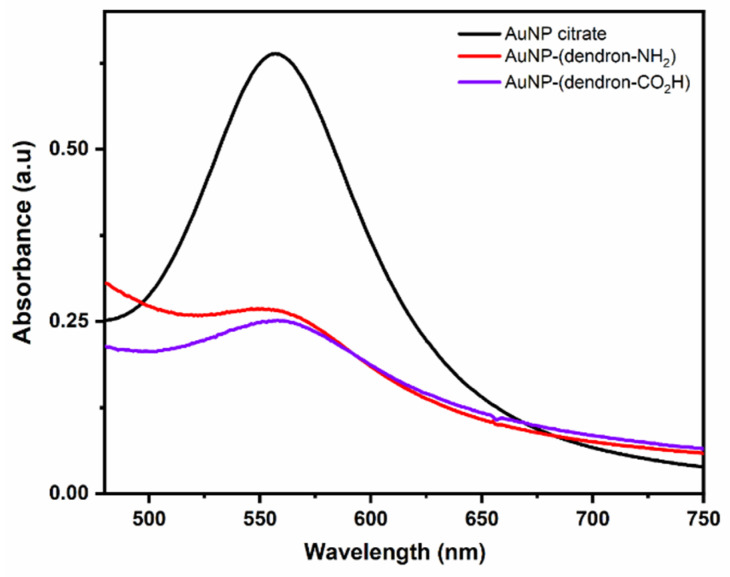
Absorption spectra in the visible region for all AuNPs at 100 μM in PBS 0.1 M.

Developing photothermally active nanomaterials to absorb radiation and generate a local hyperthermic effect is a compelling demand. The plasmonic absorption is not only associated with colloidal stability but also with the photothermal response of metallic nanoparticles. This strategy has been extensively employed for destroying cancer cells, but so far, only to a minimal extent to tackle pathogenic microorganisms. To address this question, we measured the UV-vis absorption of citrate-stabilized and both dendronized AuNPs at the range of 400–800 nm (Figure 4). The UV-vis spectra of citrate- and dendron-stabilized AuNPs reveals the prominent plasmonic band at 560 nm. These intense absorption bands arose from the collective resonant oscillation of the free electrons of the conduction band of the metal [62]. It is important to note that the plasmon band did not shift upon ligand exchange, confirming that the synthesis of the dendronized AuNPs was efficient and did not promote aggregation of the AuNPs.

## 4. Conclusions

Two charged polyamide dendrons were designed and synthesized to decorate AuNPs. Amine- and carboxyl-terminated dendrons were both obtained in relatively high yields (38–40%), presenting water solubility, terminal charged groups, and biocompatibility. The functionalization of AuNPs afforded high colloidal stability attested by DLS, SEM, and UV-vis spectroscopy. Notably, the samples were prepared by only vortexing without further energy to disperse the particles. The DLS technique and SEM showed that the dendronized AuNPs are well dispersed without signal of aggregation or sedimentation. Furthermore, the SEM images revealed a contrast between the organic capping and the metallic portion of the dendronized particles, corroborating the mechanism of nanoparticle functionalization. The synergistic contributions of dendrons with charged termini and AuNPs resulted in promising nanomaterials to tackle antimicrobial resistance. In vitro tests on the antimicrobial activity of these materials against several types of bacteria (Gram-positive and Gram-negative) are currently being carried out.

## Figures and Tables

**Table 1:** Values of zeta potential, hydrodynamic diameter, and PDI obtained by DLS for the AuNPs measured in ultrapure water at 1 mg/mL.

	Zeta Potential (mV ± SD)	Diameter (nm ± SD)	PDI (PDI ± SD)
AuNP–citrate	−49 ± 9	60 ± 2	0.06 ± 0.01
AuNP–(dendron-NH_2_)	27 ± 6	109 ± 1	0.19 ± 0.09
AuNP–(dendron-CO_2_H)	−31 ± 8	80 ± 1	0.05 ± 0.01

## Data Availability

Data is contained within the article or Supplementary Material.

## Supplementary Materials

The following supporting information can be downloaded at: https://www.mdpi.com/article/10.3390/nano12152610/s1, Figures S1–S35: NMR and mass spectra for all compounds.
