# Supplementary material for: Synthesis and Characterization of Dendronized Gold Nanoparticles Bearing Charged Peripheral Groups with Antimicrobial Potential
Source: Nanomaterials (Basel). 2022 Jul 29;12(15):2610. doi: 10.3390/nano12152610 (PMC9370457; doi:10.3390/nano12152610)
Supplement: Supplementary file 1 [file nanomaterials-12-02610-s001.zip › nanomaterials-1794582-supplementary.pdf]

Synthesis and Characterization of Dendronized Gold Nanoparticles Bearing Charged Peripheral Groups with Antimicrobial Potential

Gabriel Perli [1], Diego L. Bertuzzi [1], Dênio E. P. Souto [1,2], Miguel D. Ramos [1], Carolyne B. Braga [1], Samile B. Aguiar [1] and Catia Ornelas [1,*]

[1] Institute of Chemistry, University of Campinas, Campinas 13083-970, SP, Brazil; perligabriel@gmail.com (G.P.); diego.bertuzzi@hotmail.com (D.L.B.); denio.souto@ufpr.br (D.E.P.S.); migueljr.qma@gmail.com (M.D.R.); carolbbraga7@gmail.com (C.B.B.); samilede17@gmail.com (S.B.A.)

[2] Laboratorio de Espectrometria, Sensores e Biossensores, Departamento de Quimica, Universidade Federal do Paraná (UFPR), Curitiba 81531-980, PR, Brazil

* Correspondence: catiaornelas@catiaornelaslab.com

1. NMR and Mass Spectra for all Compounds

1.2. Compound 2

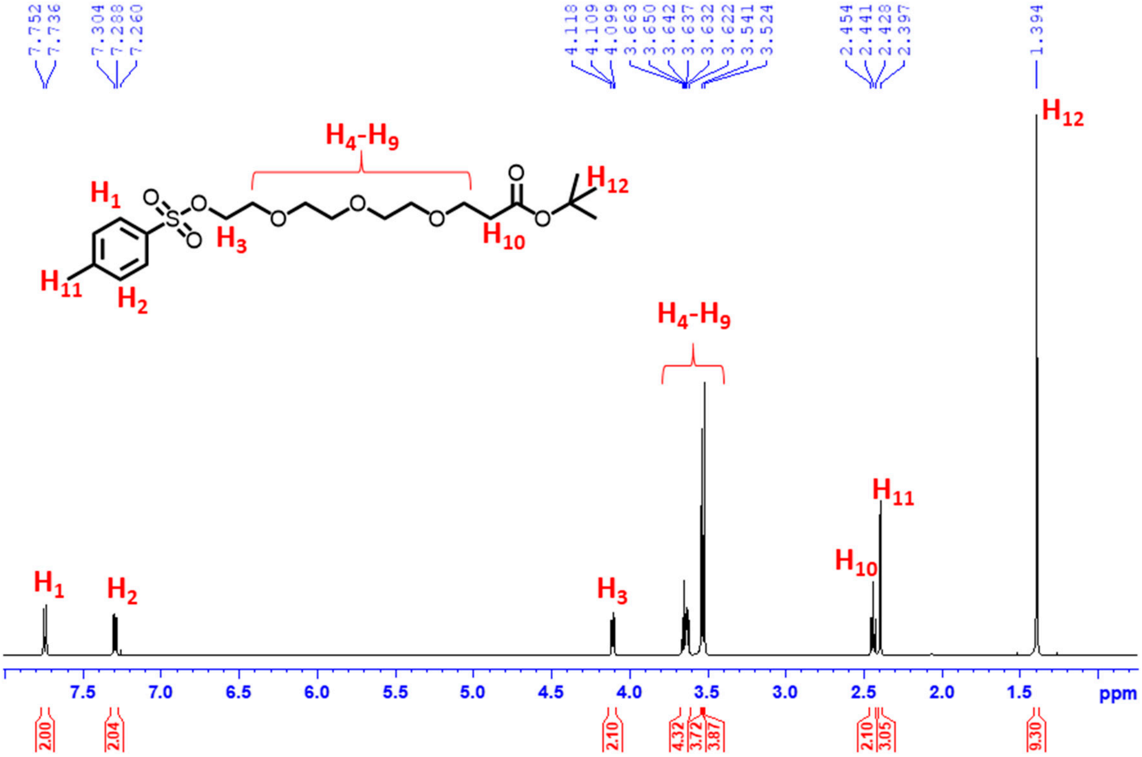

Figure S1. ^{1}H NMR spectrum of compound **2** (500 MHz, $CDCl_3$, 25 ºC).

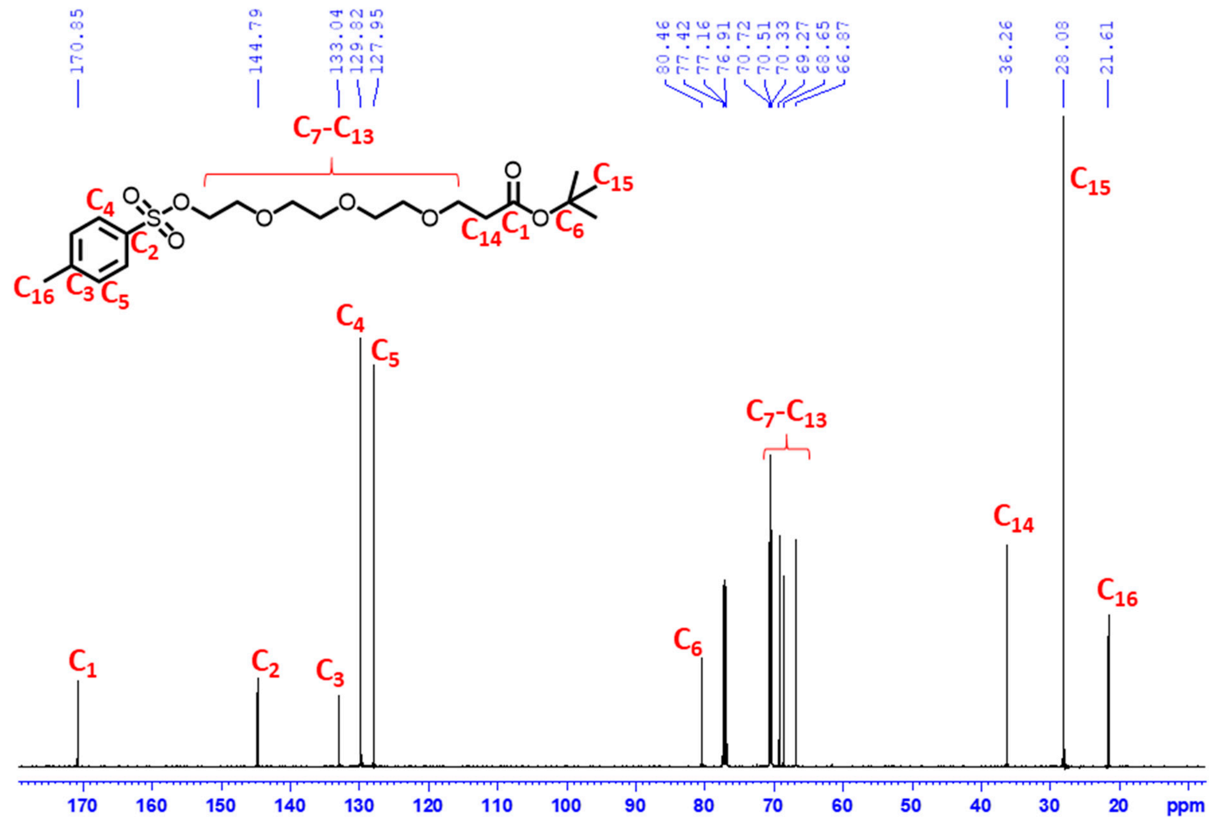

Figure S2. ^{13}C NMR spectrum of compound **2** (500 MHz, CDCl3, 25 ºC).

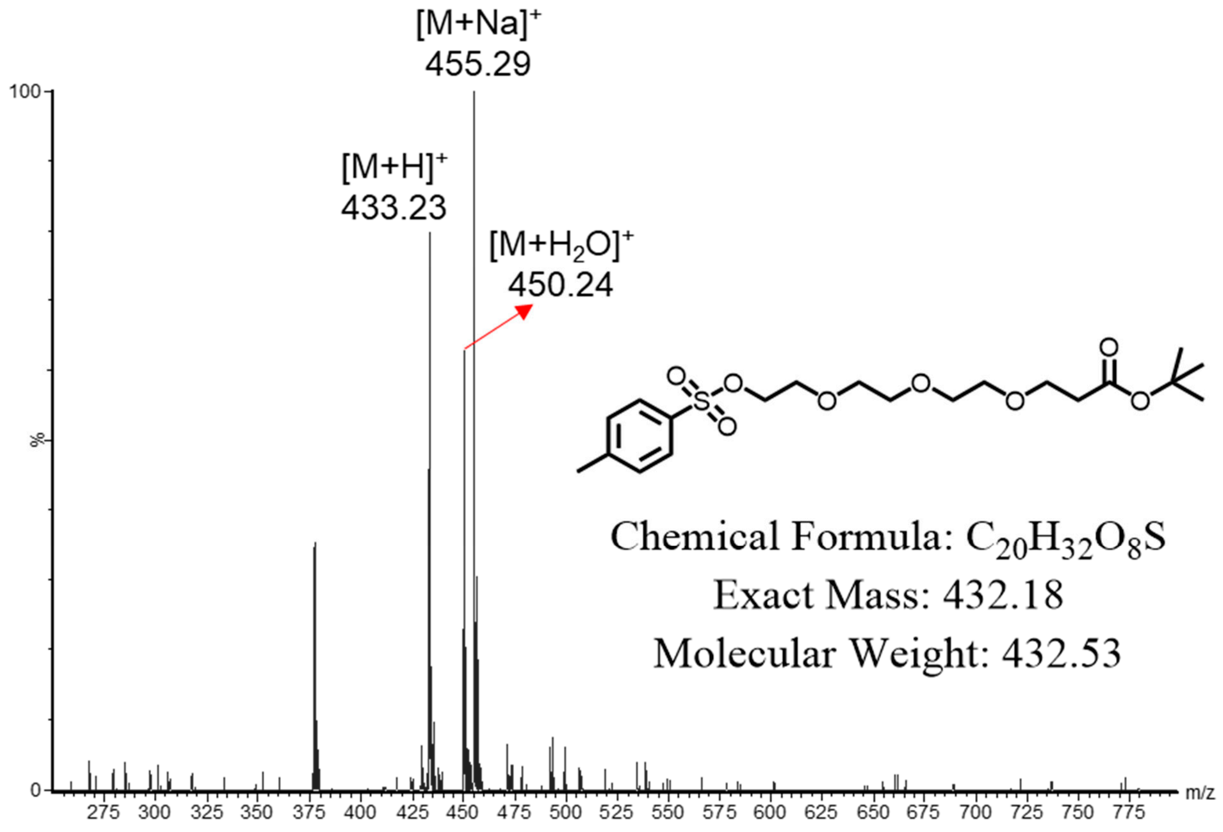

Figure S3. ESI-QTOF mass spectrum of compound **2**.

1.3. Compound 3

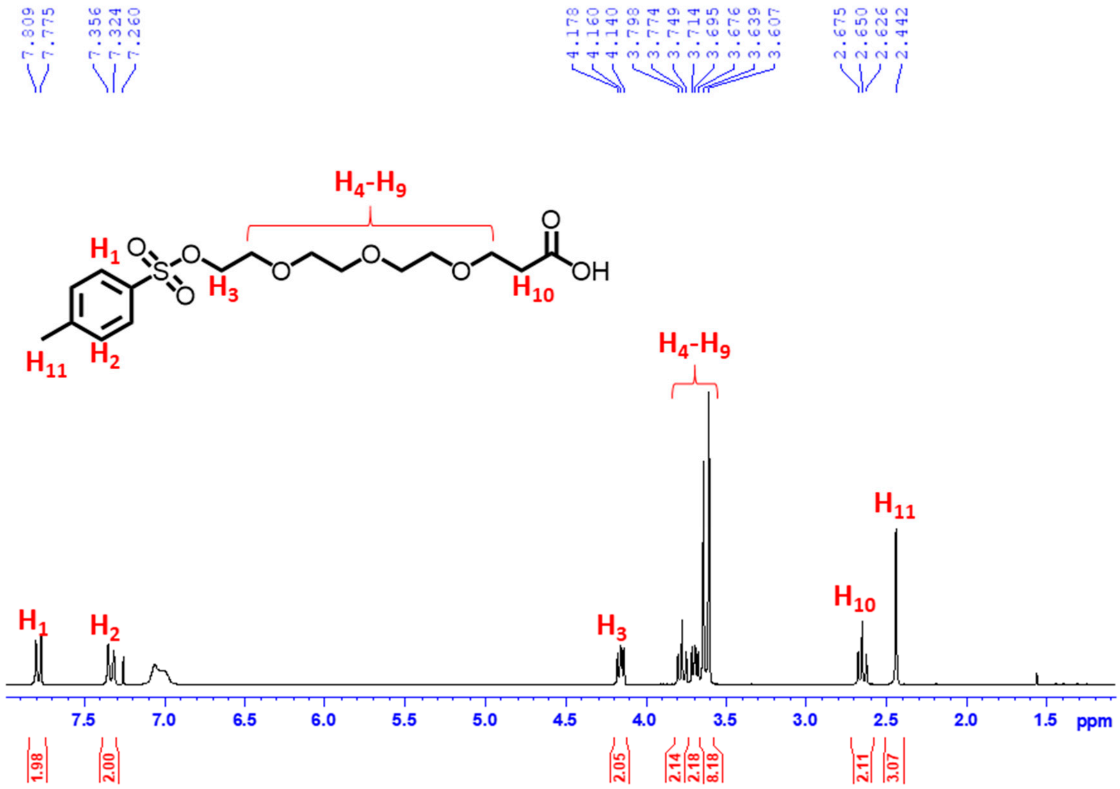

Figure S4. ^{1}H NMR spectrum of compound **3** (500 MHz, $CDCl_3$, 25 ºC).

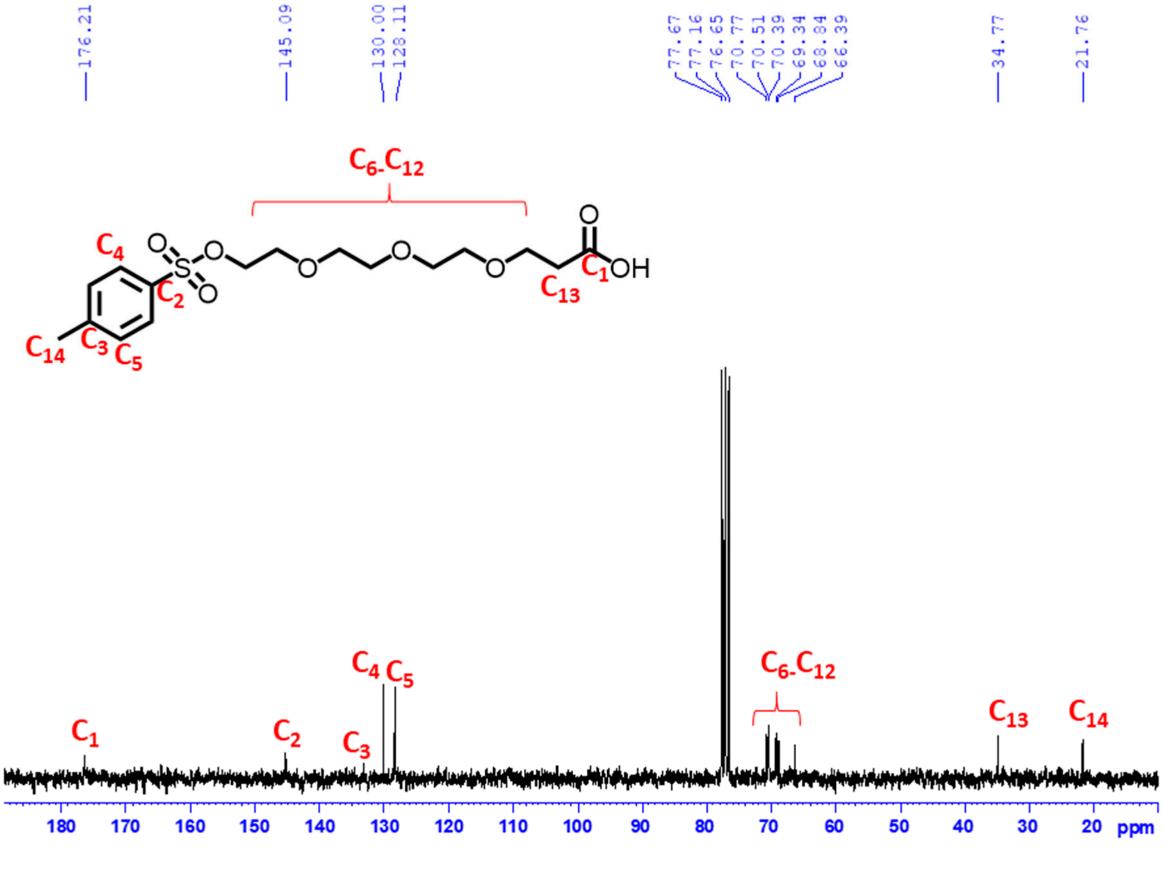

Figure S5. ^{13}C NMR spectrum of compound **3** (500 MHz, $CDCl_3$, 25 ºC).

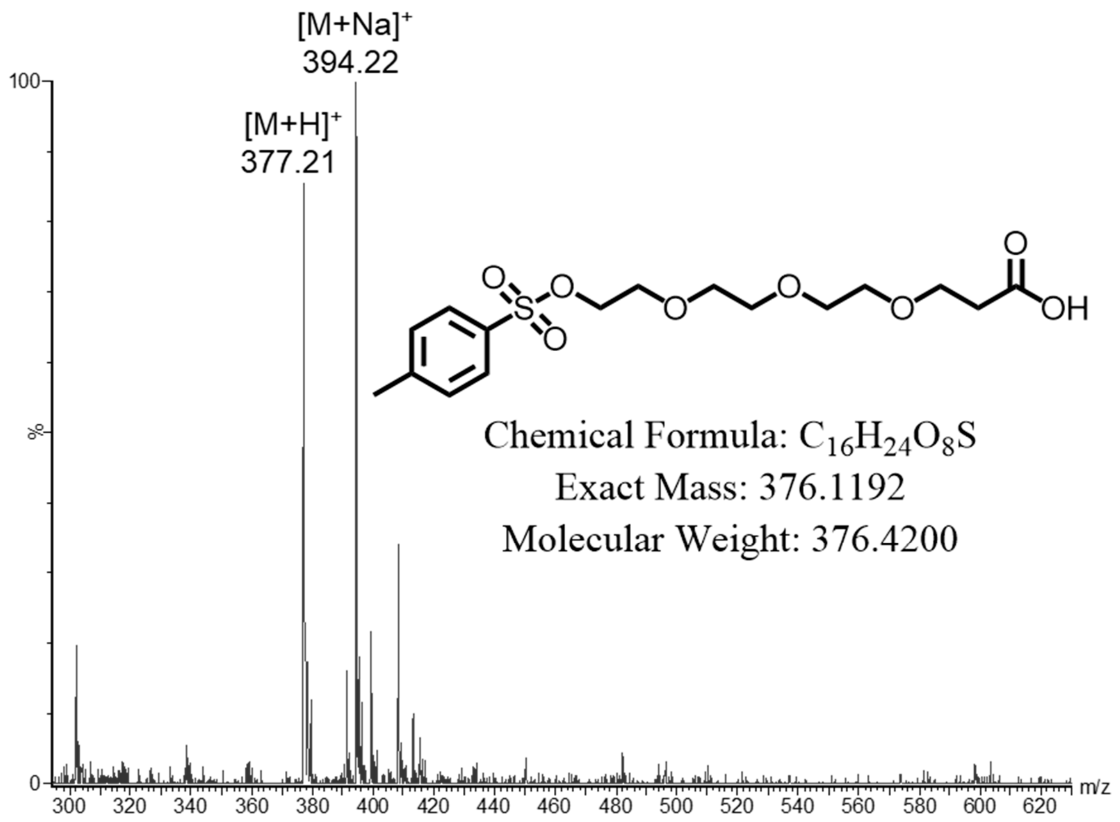

Figure S6. ESI-QTOF mass spectrum of compound **3**.

1.4. Compound 4

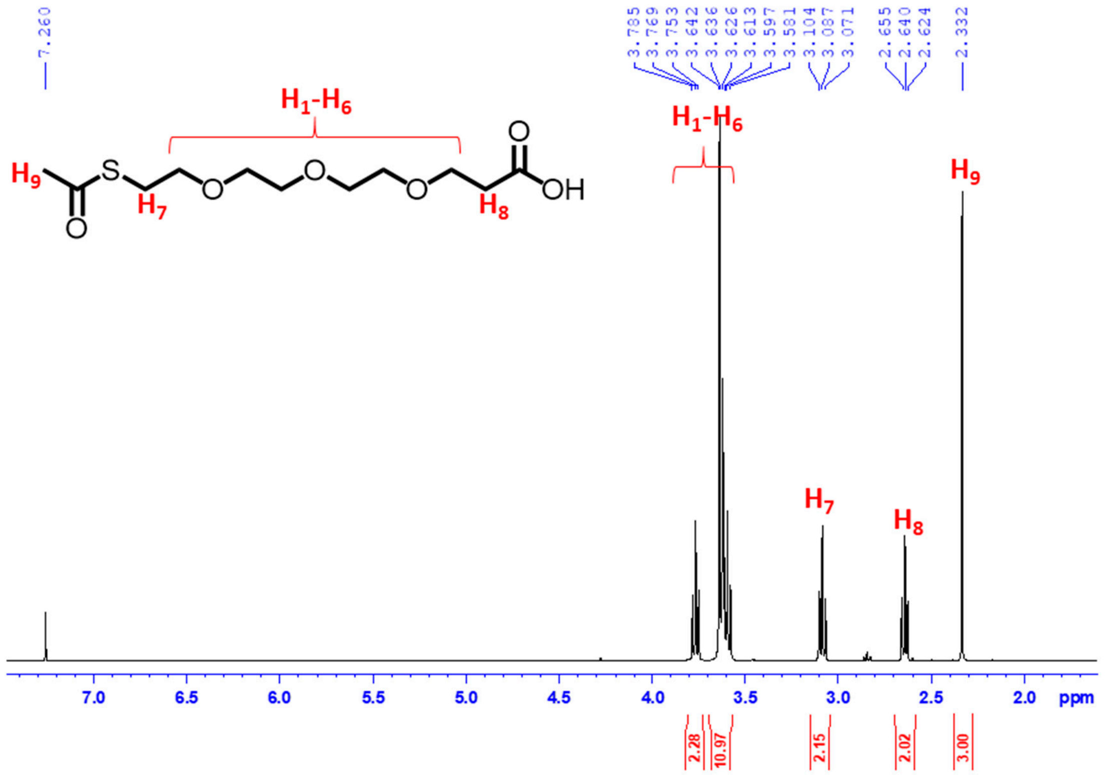

Figure S7. ^{1}H NMR spectrum of compound **4** (500 MHz, $CDCl_3$, 25 ºC).

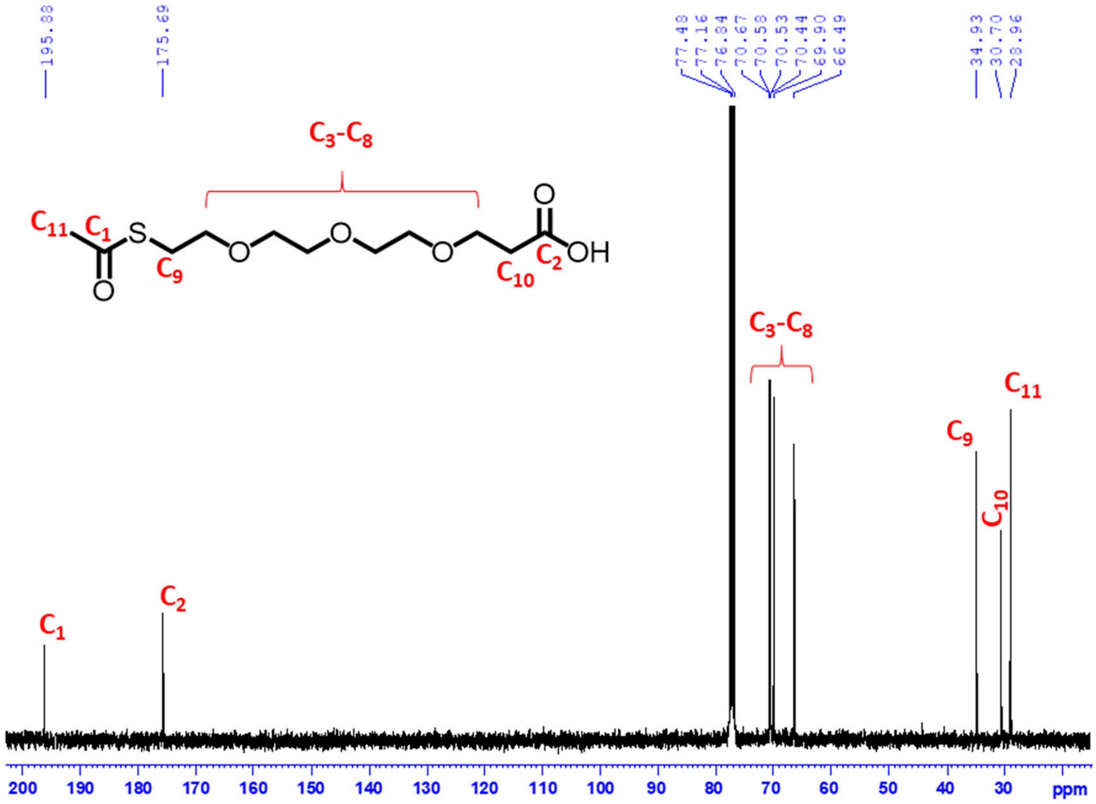

Figure S8. ^{13}C NMR spectrum of compound **4** (500 MHz, $CDCl_3$, 25 ºC).

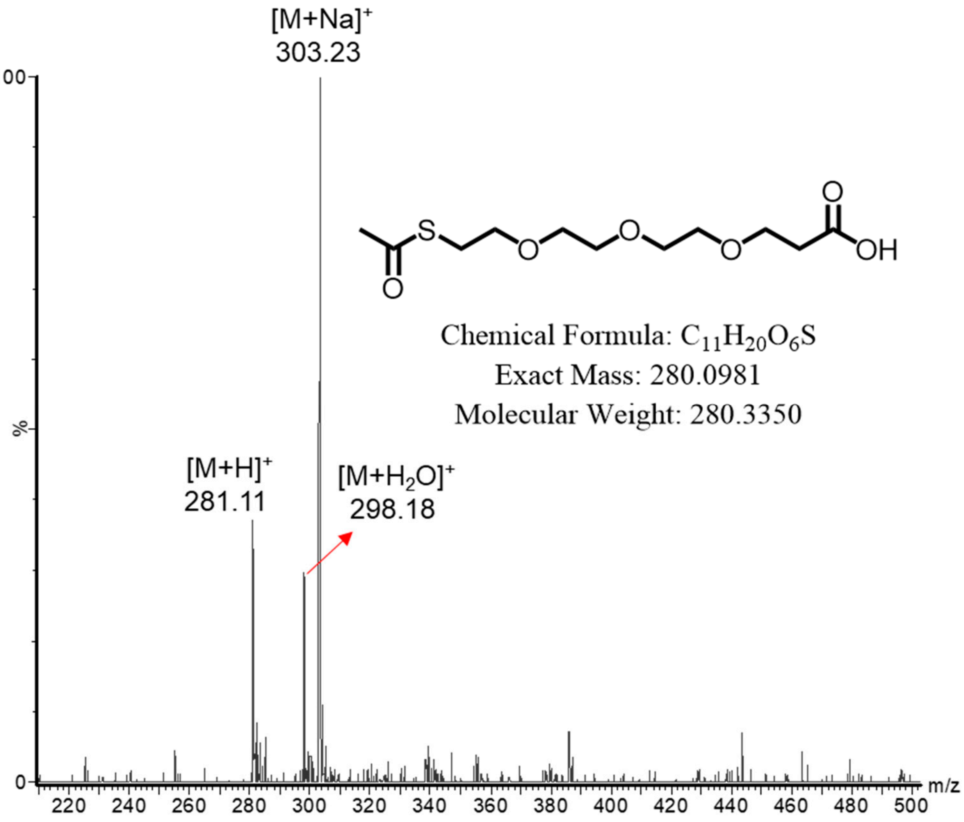

Figure S9. ESI-QTOF mass spectrum of compound **4.**

1.5. Compound 5

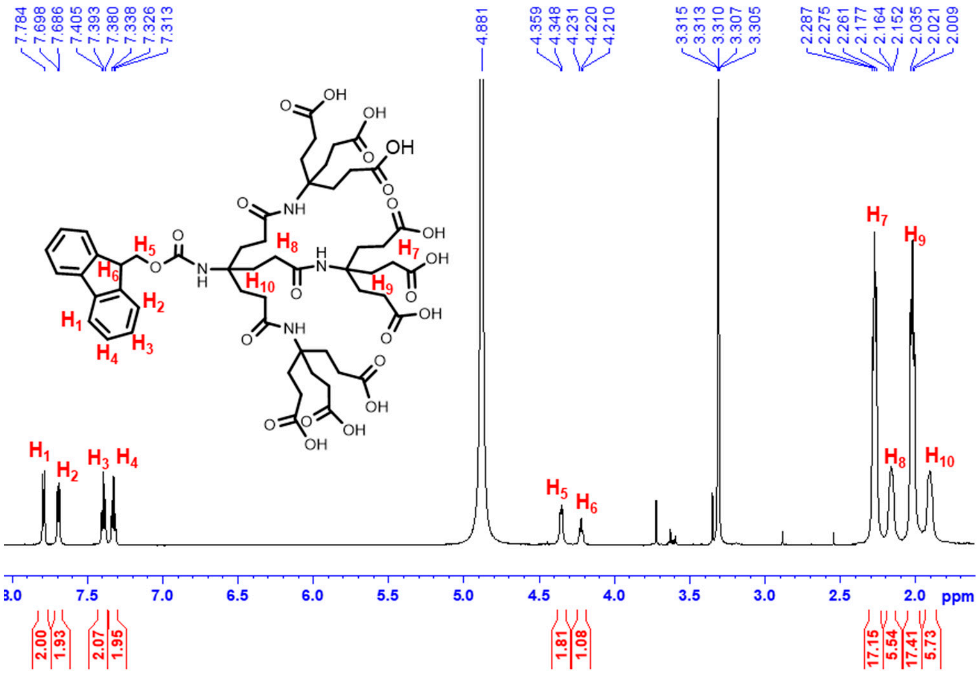

Figure S10. ^{1}H NMR spectrum of compound **5** (600 MHz, MeOD, 25 ºC).

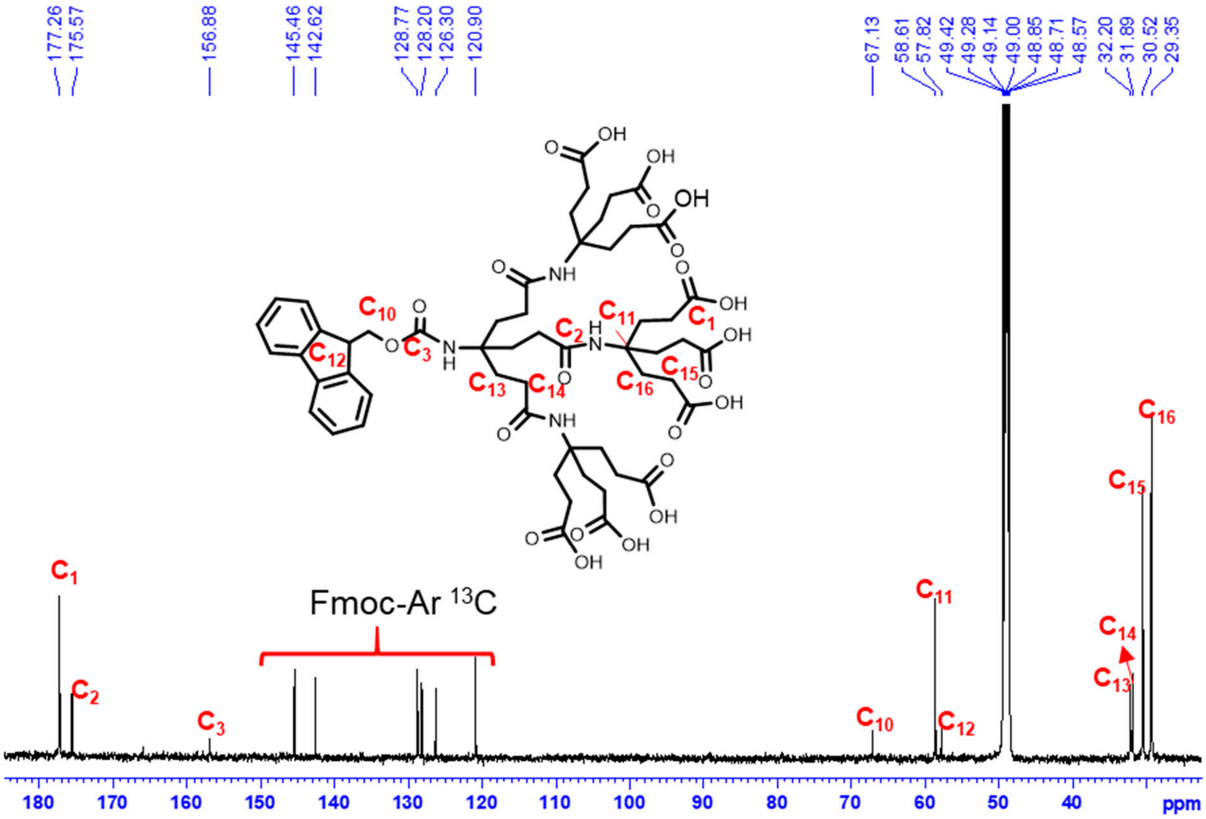

Figure S11. ^{13}C NMR spectrum of compound **5** (600 MHz, MeOD, 25 ºC).

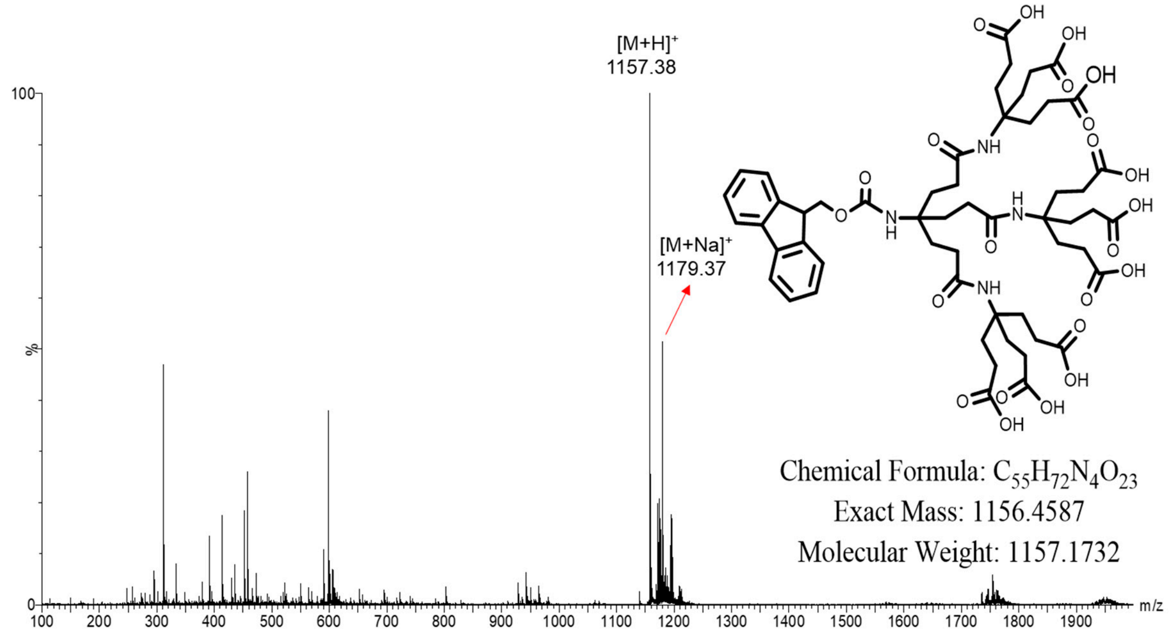

Figure S12. ESI-QTOF mass spectrum of compound **5**.

1.6. Compound 6

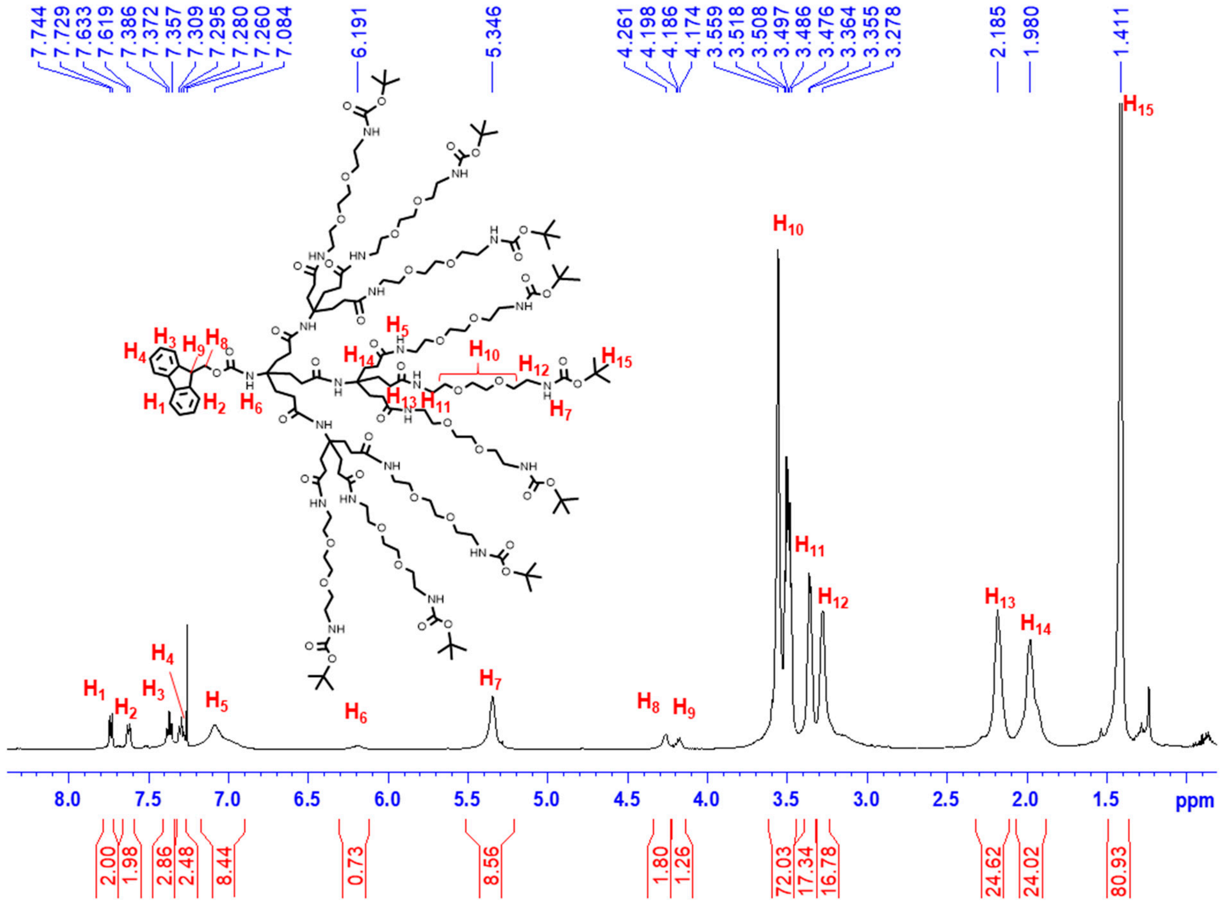

Figure S13. ^{1}H NMR spectrum of compound **6** (600 MHz, $CDCl_3$, 25 ºC).

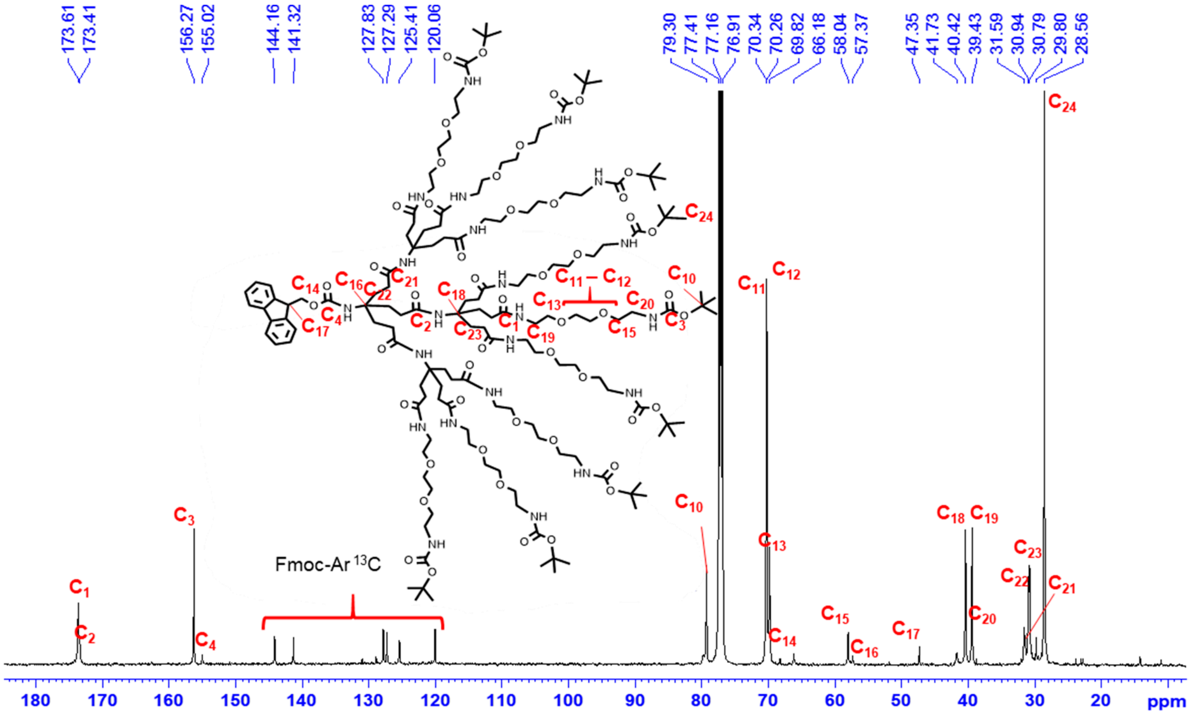

Figure S14. ^{13}C NMR spectrum of compound **6** (600 MHz, $CDCl_3$, 25 ºC).

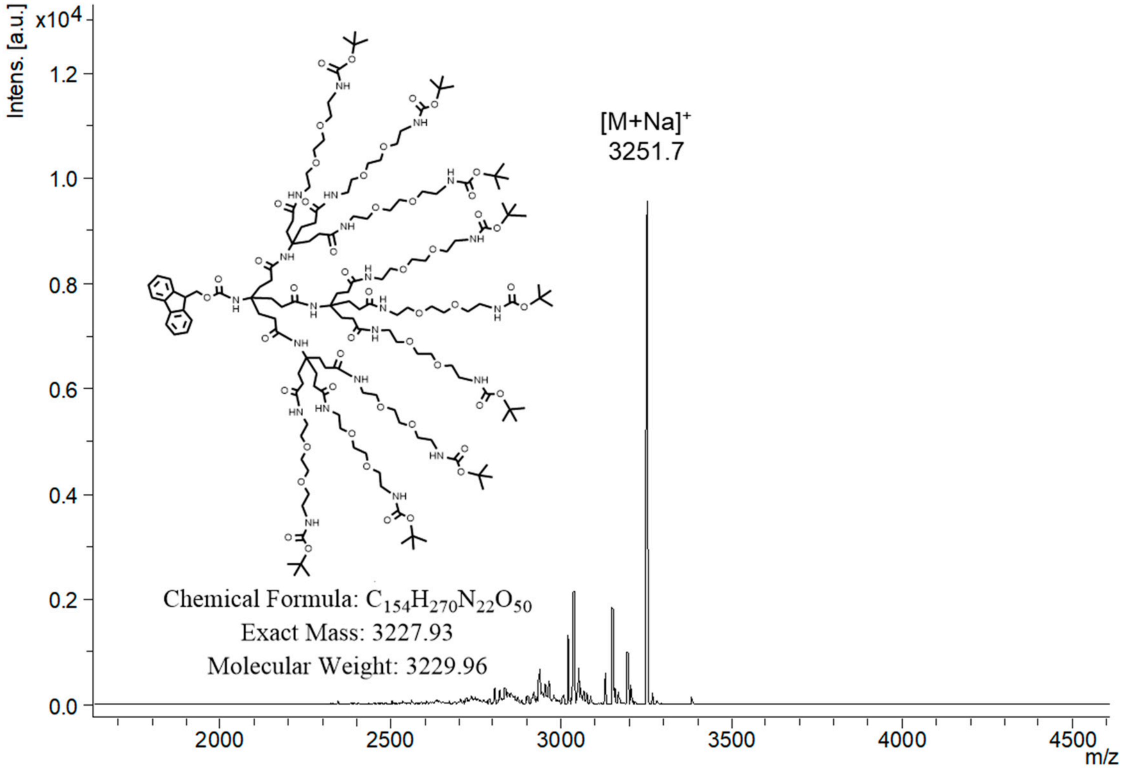

Figure S15. MALDI-TOF mass spectrum of compound **6**.

1.7. Compound 7

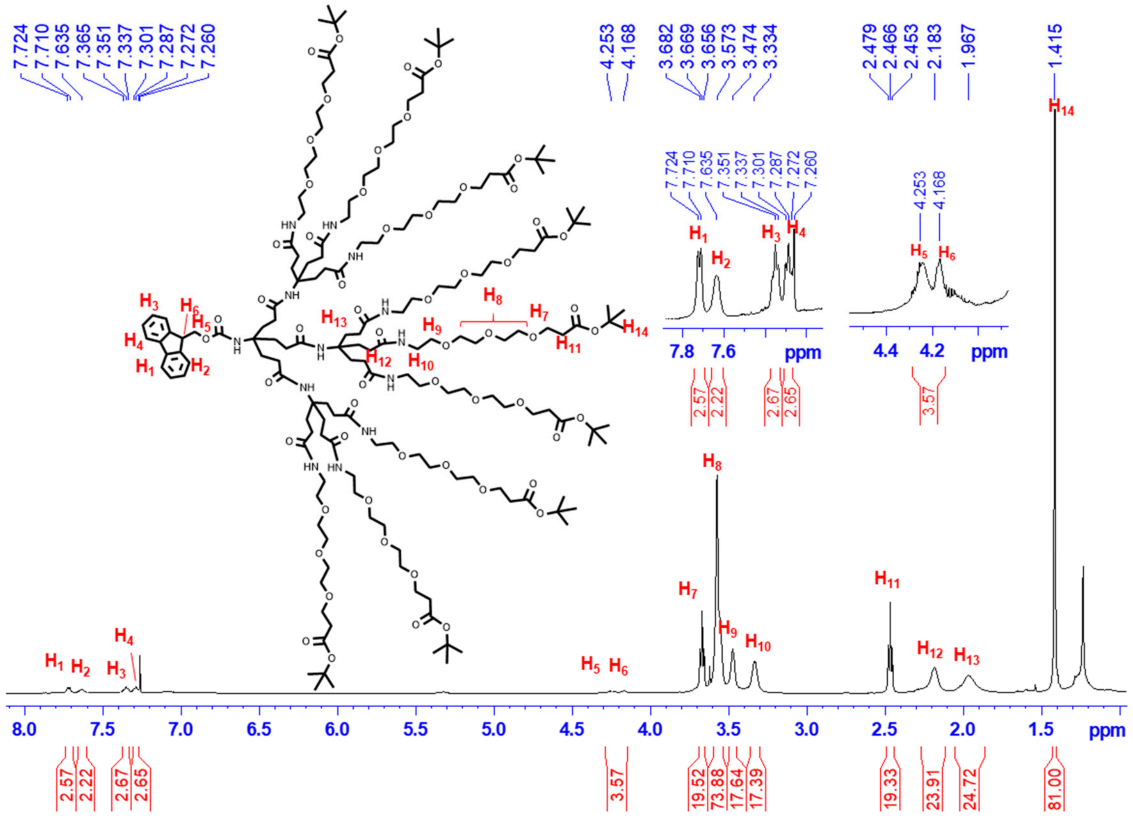

Figure S16. ^{1}H NMR spectrum of compound **7** (600 MHz, $CDCl_3$, 25 ºC).

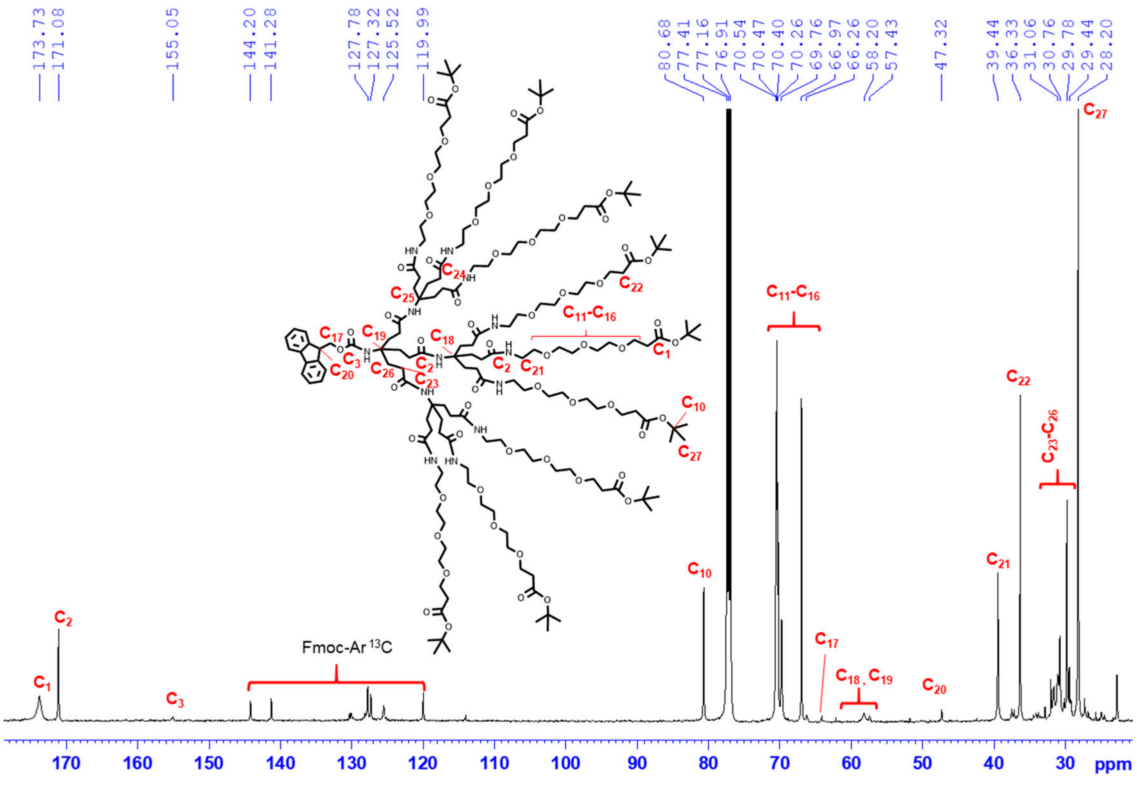

Figure S17. ^{13}C NMR spectrum of compound **7** (600 MHz, $CDCl_3$, 25 ºC).

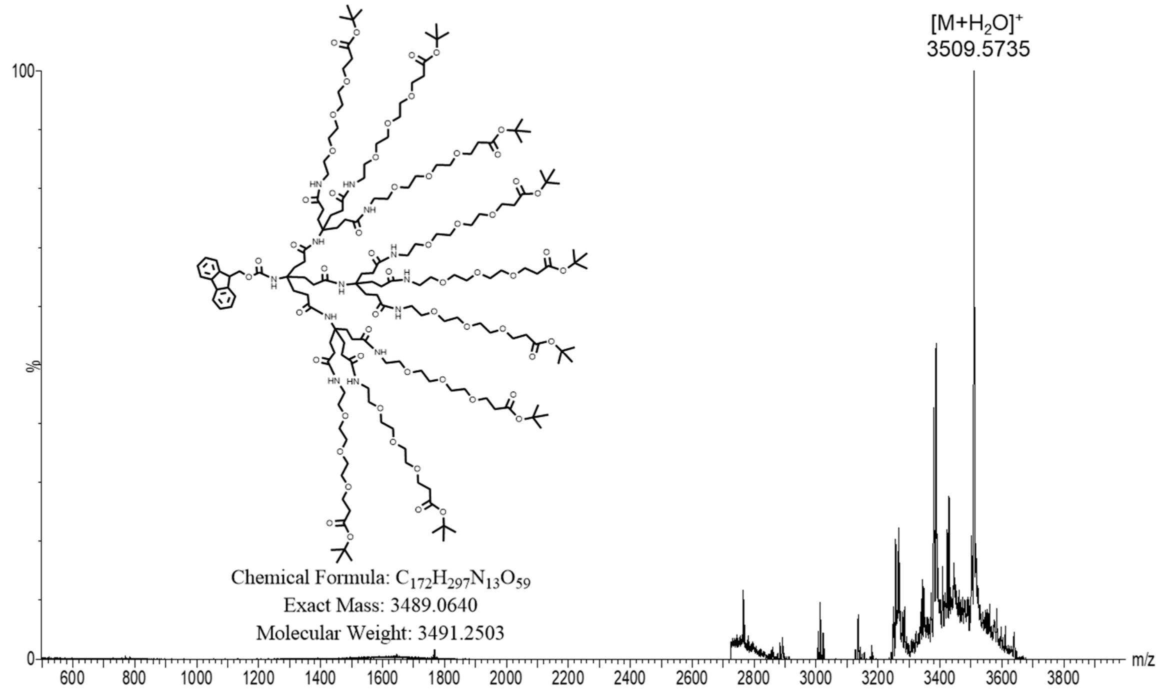

Figure S18. MALDI-TOF mass spectrum of compound **7**.

1.8. Compound 8

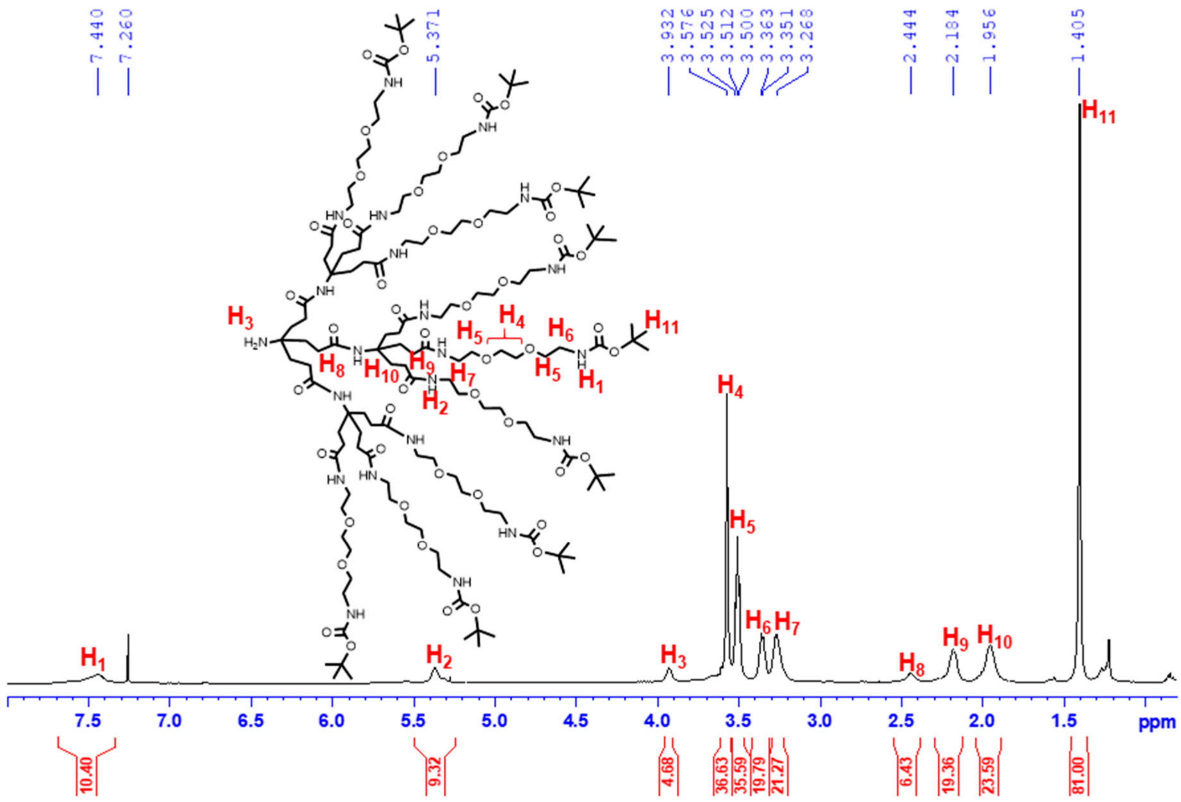

Figure S19. ^{1}H NMR spectrum of compound **8** (600 MHz, $CDCl_3$, 25 ºC).

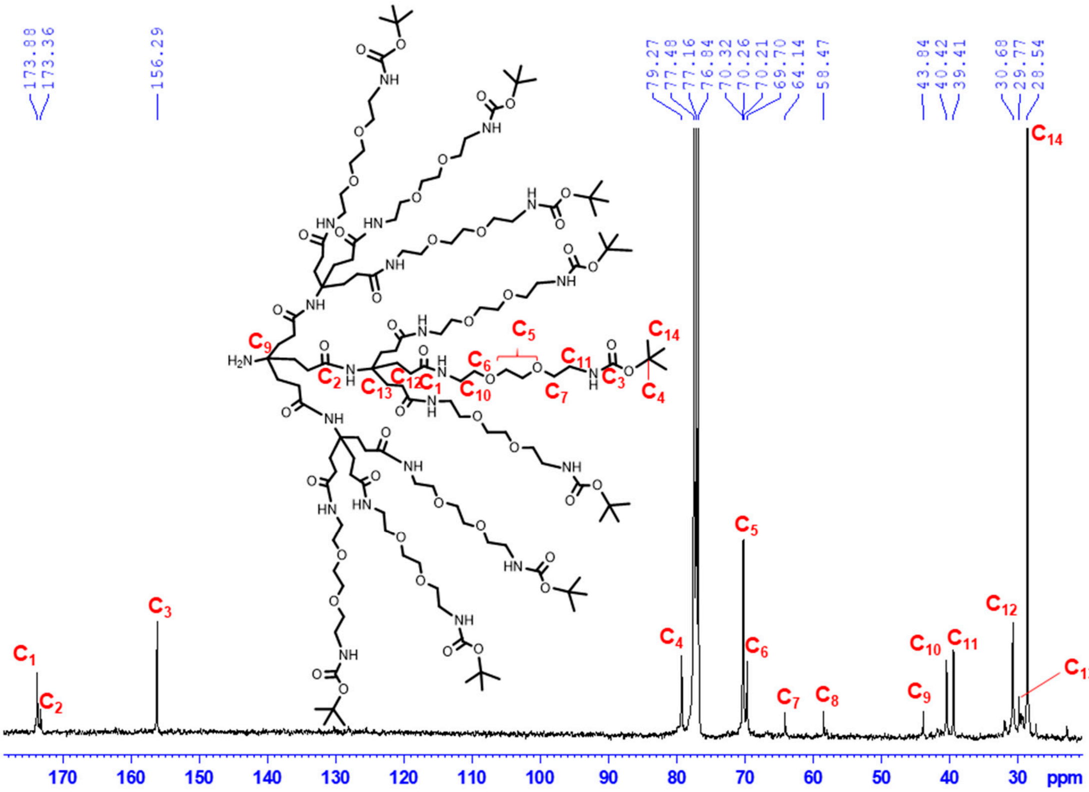

Figure S20. ^{1}H NMR spectrum of compound **8** (600 MHz, $CDCl_3$, 25 ºC).

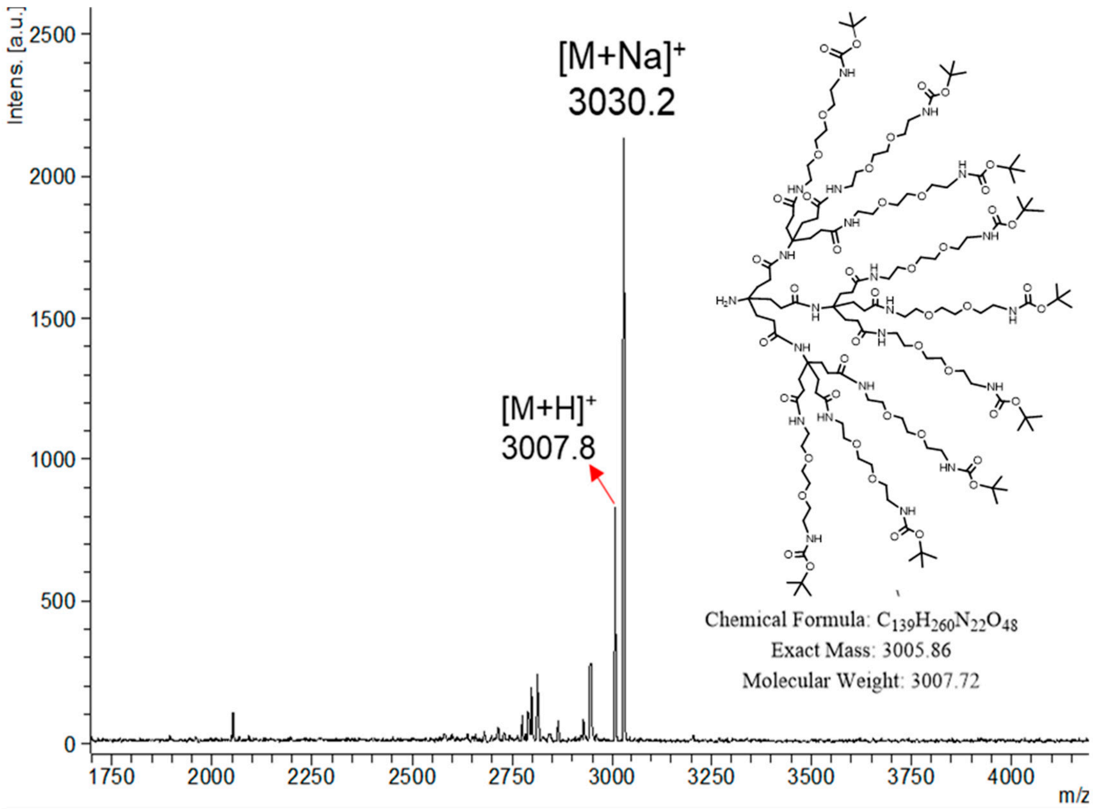

Figure S21. ESI-QTOF mass spectrum of compound **8**.

1.9. Compound 9

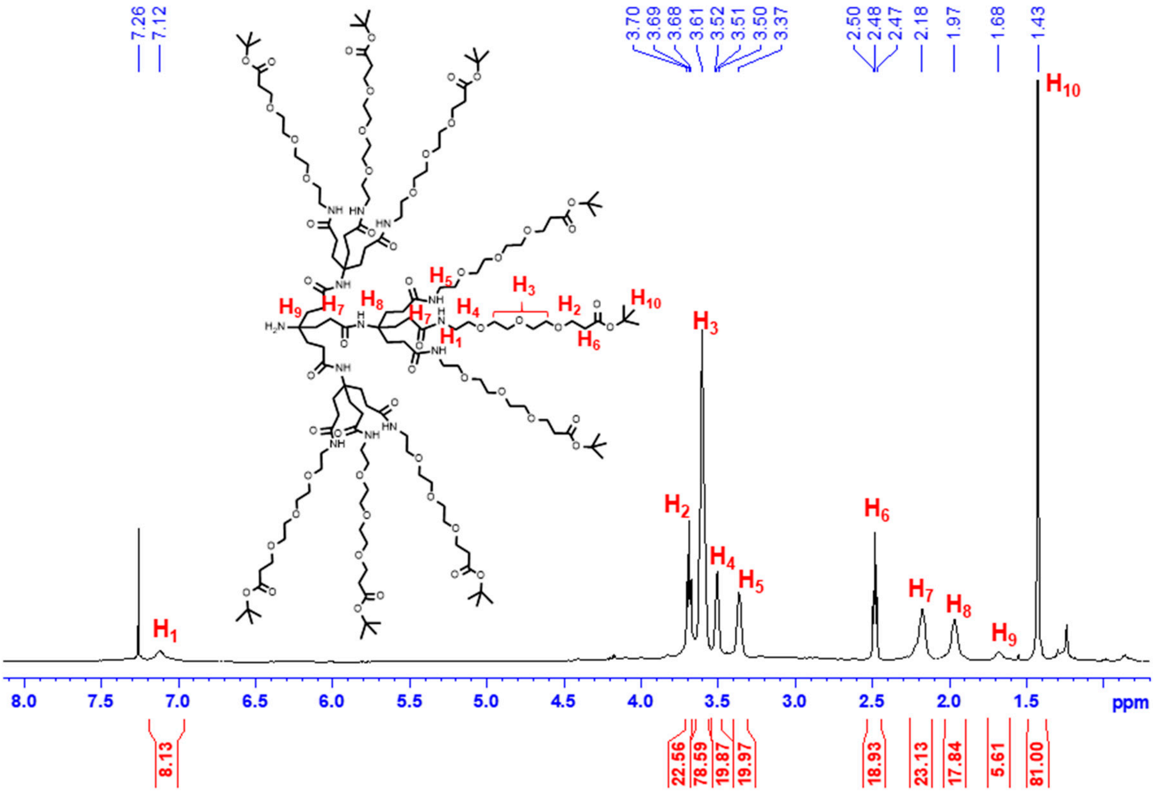

Figure S22. ^{1}H NMR spectrum of compound **9** (600 MHz, $CDCl_3$, 25 ºC).

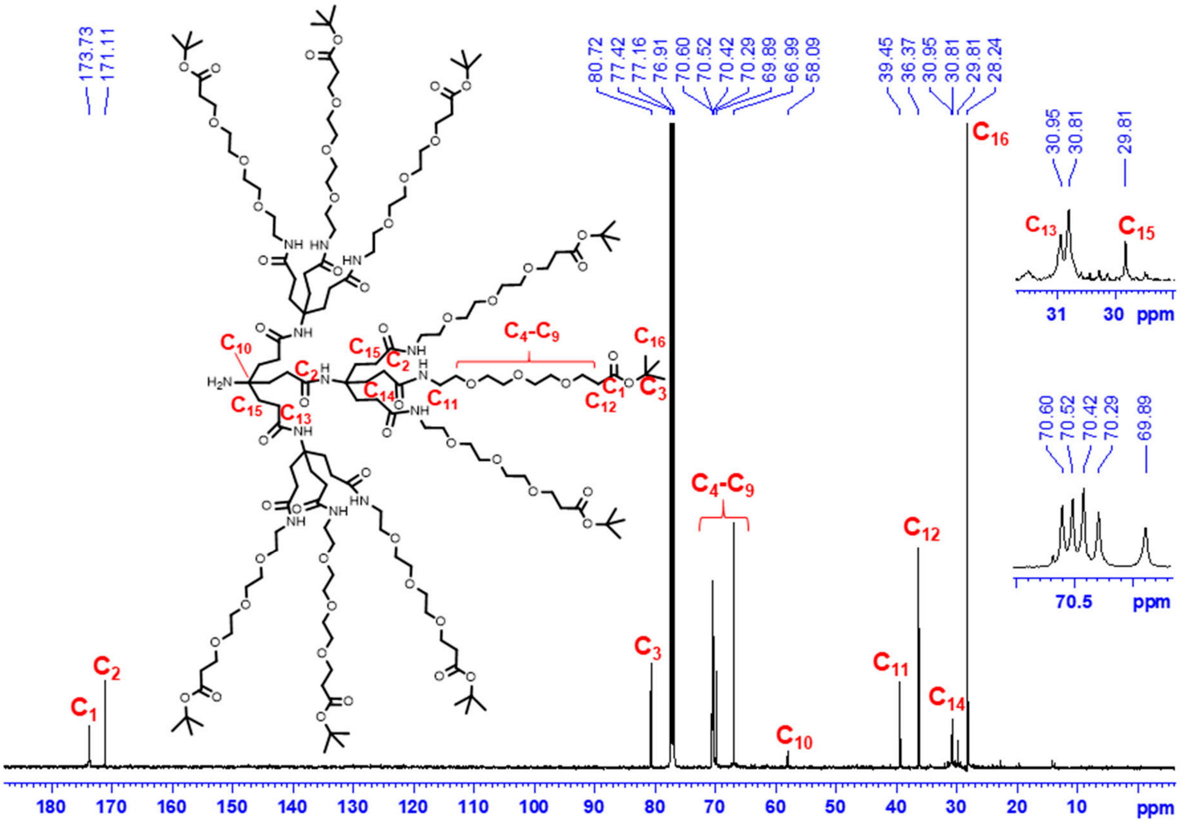

Figure S23. ^{13}C NMR spectrum of compound **9** (600 MHz, $CDCl_3$, 25 ºC).

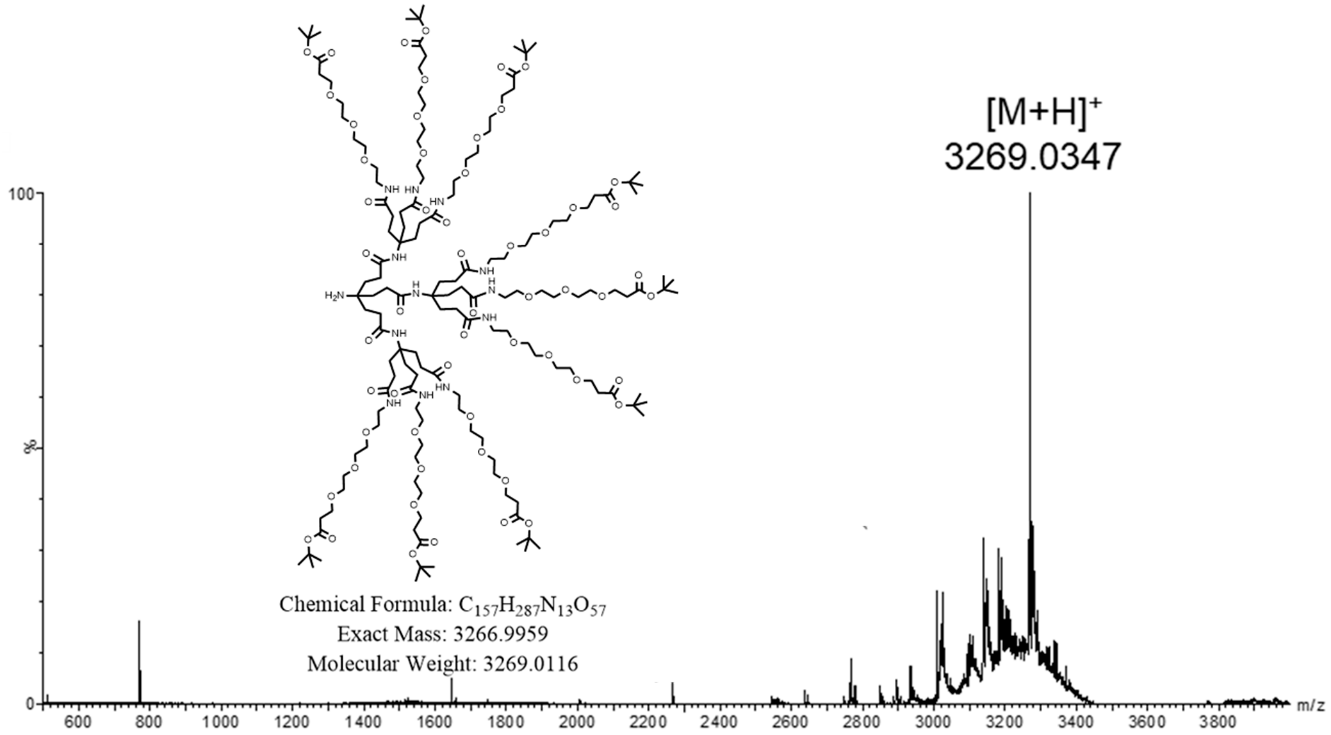

Figure S24. ESI-QTOF mass spectrum of compound **9**.

1.10. Compound 10

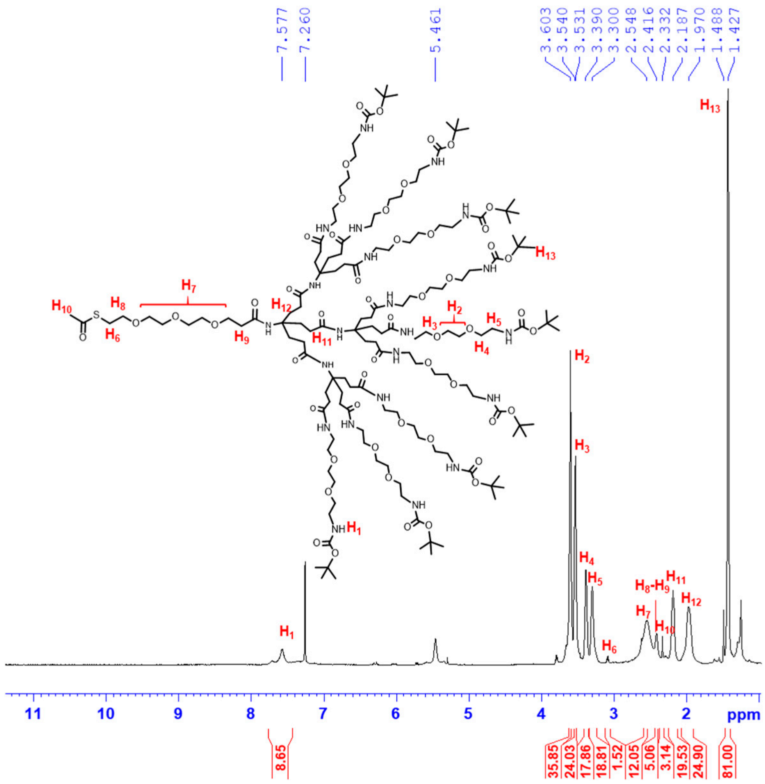

Figure S25. ^{1}H NMR spectrum of compound **10** (600 MHz, $CDCl_3$, 25 ºC).

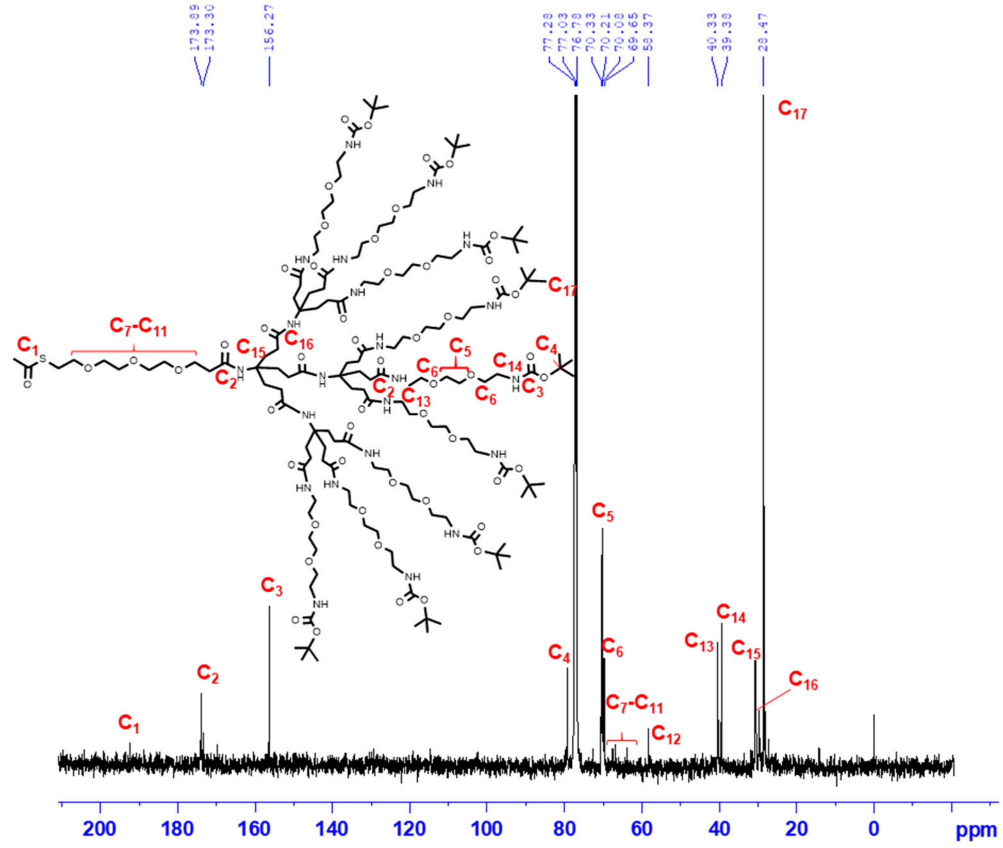

Figure S26. ^{13}C NMR spectrum of compound **10** (600 MHz, $CDCl_3$, 25 ºC).

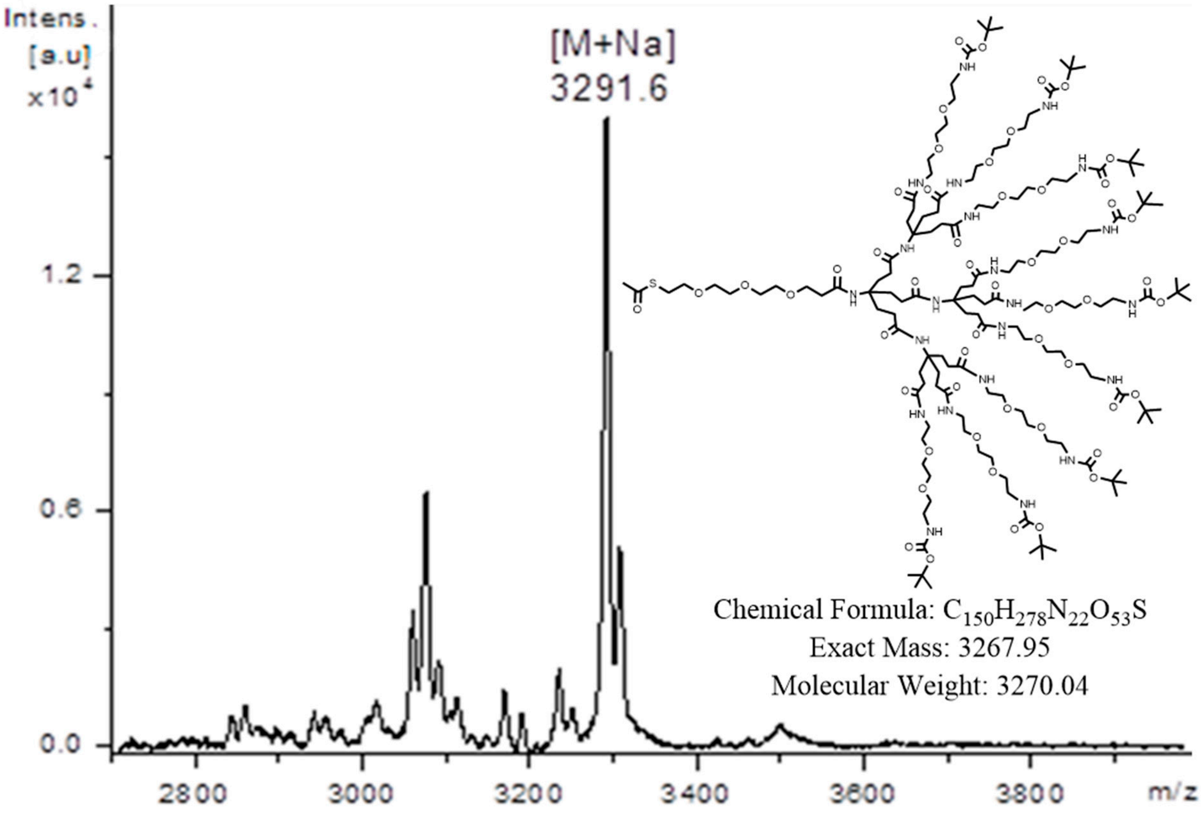

Figure S27. ESI-QTOF mass spectrum of compound **10**.

1.11. Compound 11

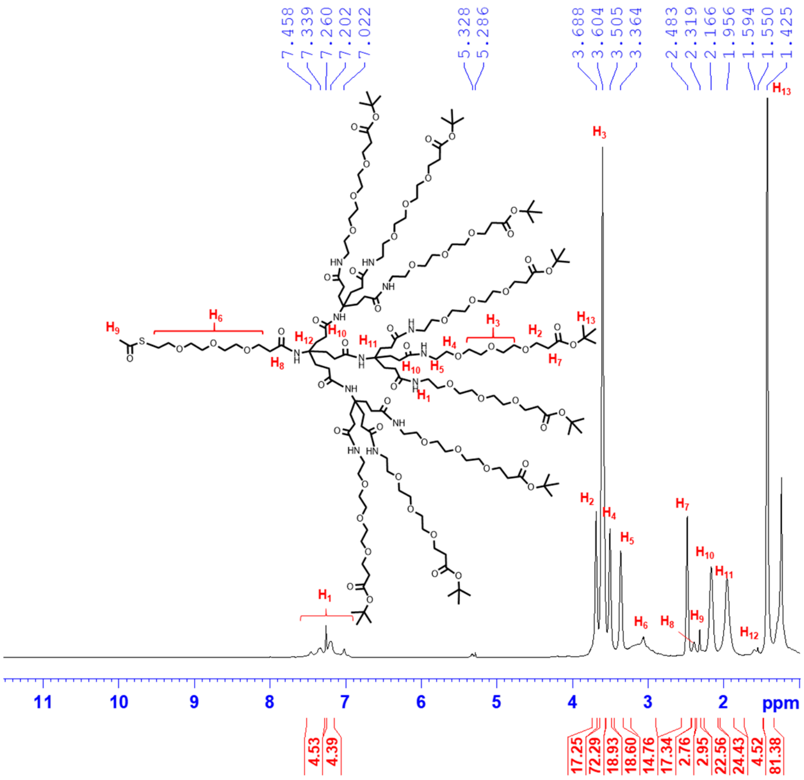

Figure S28. ^{1}H NMR spectrum of compound **11** (600 MHz, CDCl$_3$, 25 ºC).

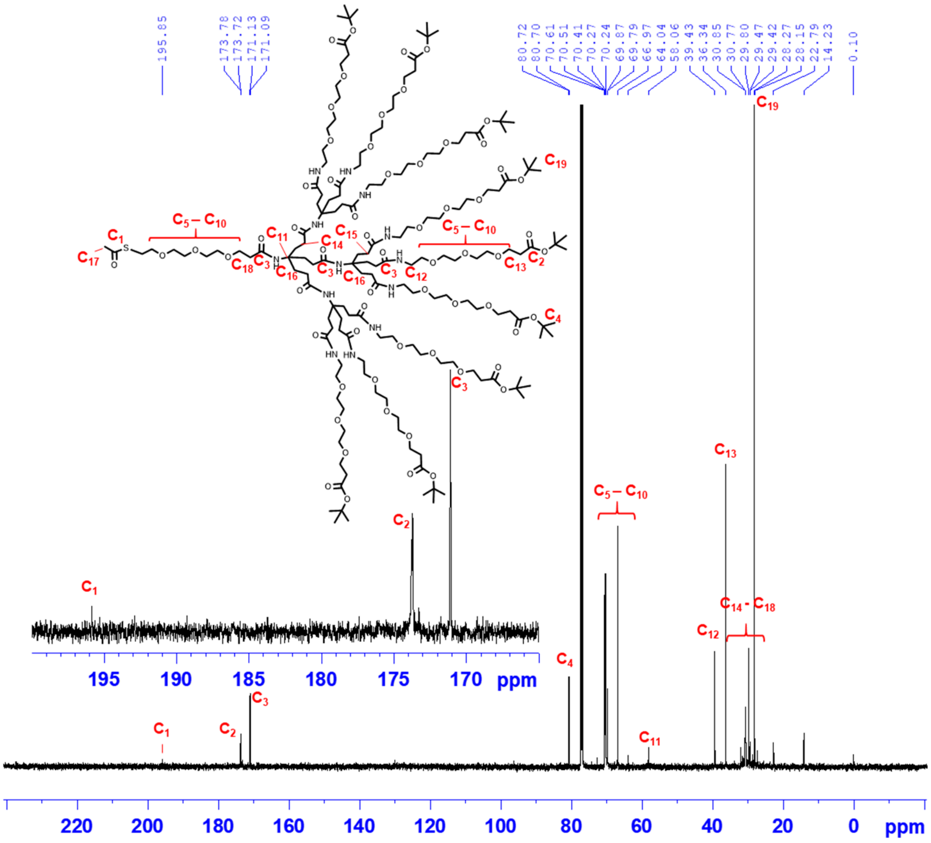

Figure S29. ^{13}C NMR spectrum of compound **11** (600 MHz, CDCl$_3$, 25 ºC).

1.12. Compound 12

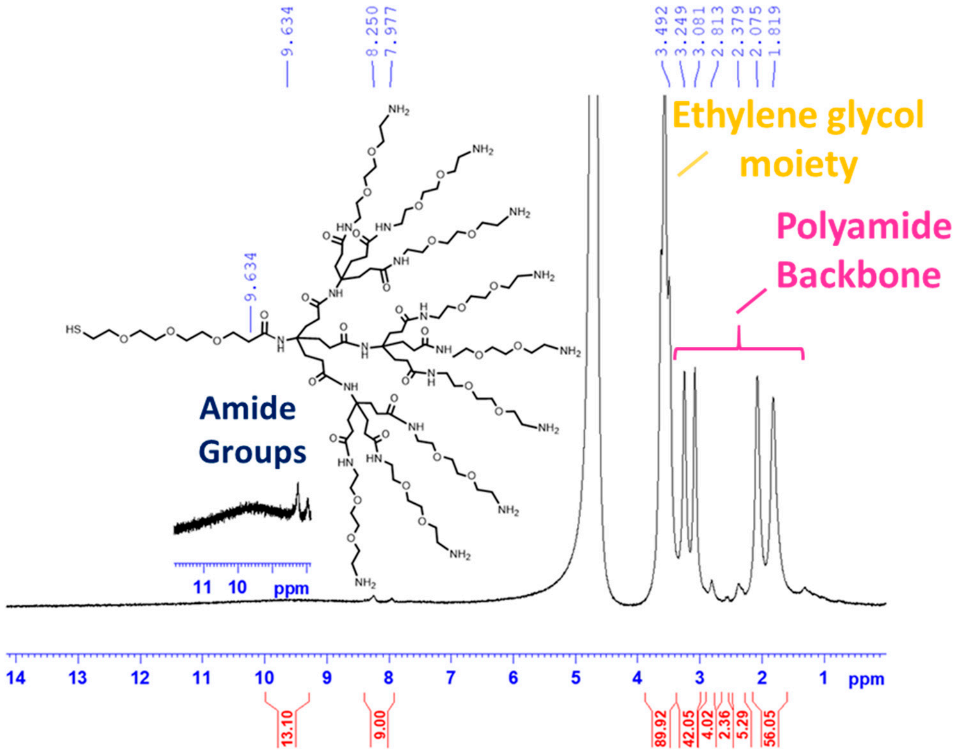

Figure S30. ^{1}H NMR spectrum of compound **12** (600 MHz, D_2O, 25 ºC).

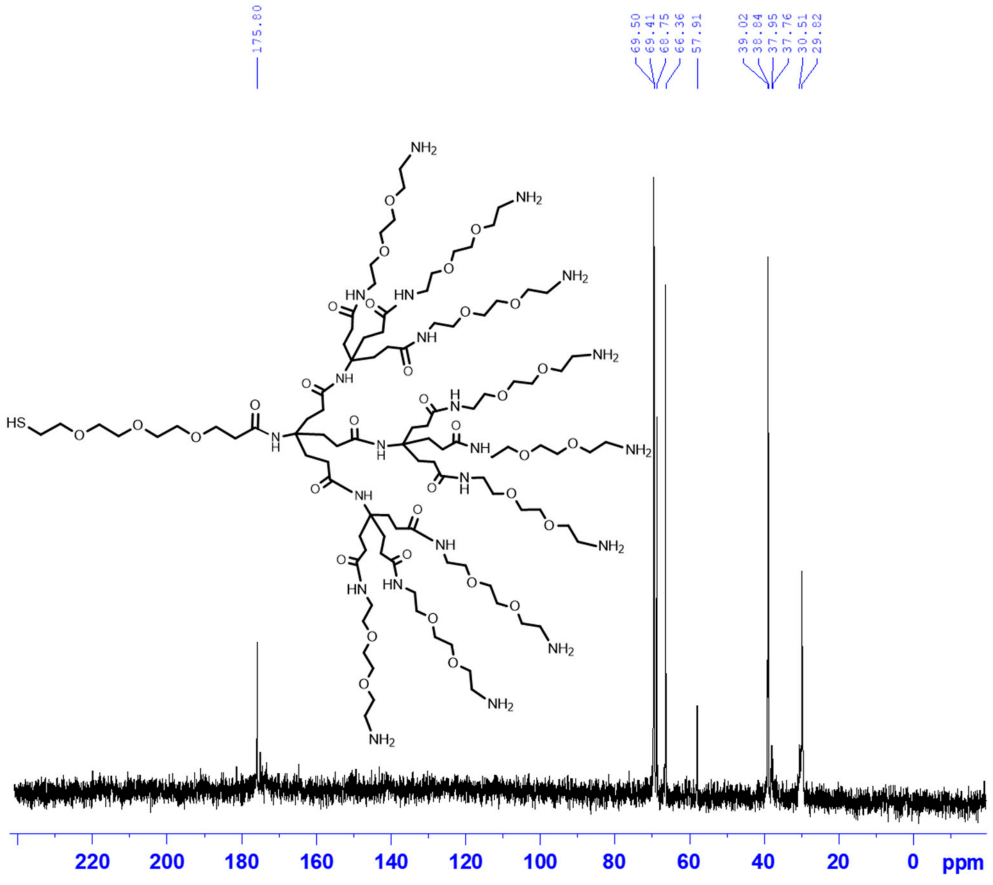

Figure S31. ^{13}C NMR spectrum of compound **12** (600 MHz, D_2O, 25 ºC).

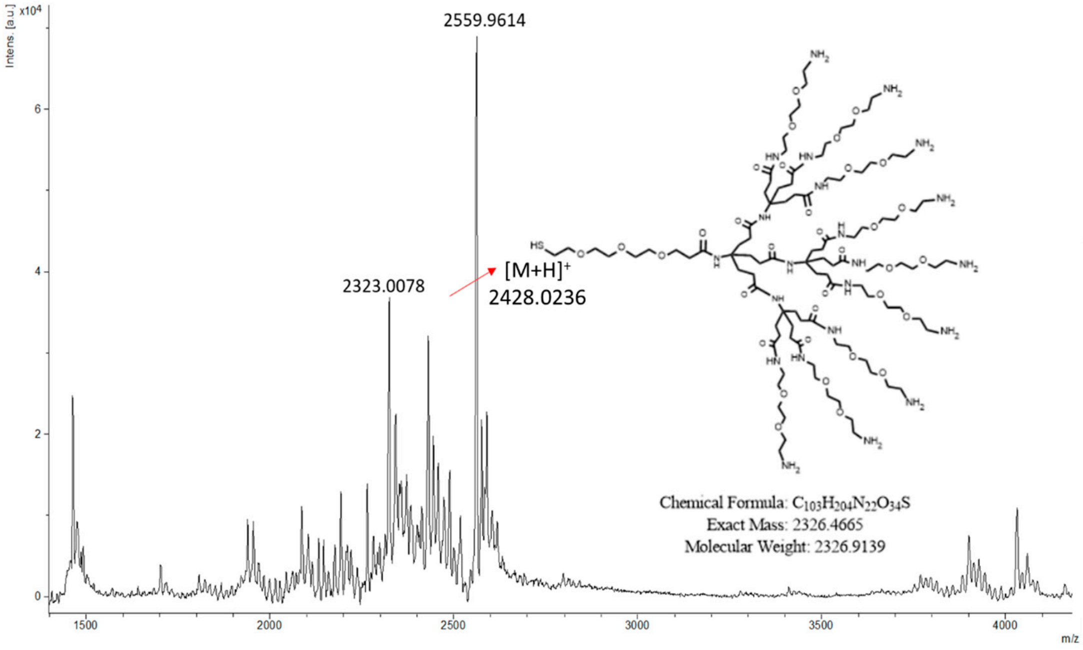

Figure S32. MALDI-TOF mass spectrum of compound **12**.

1.13. Compound 13

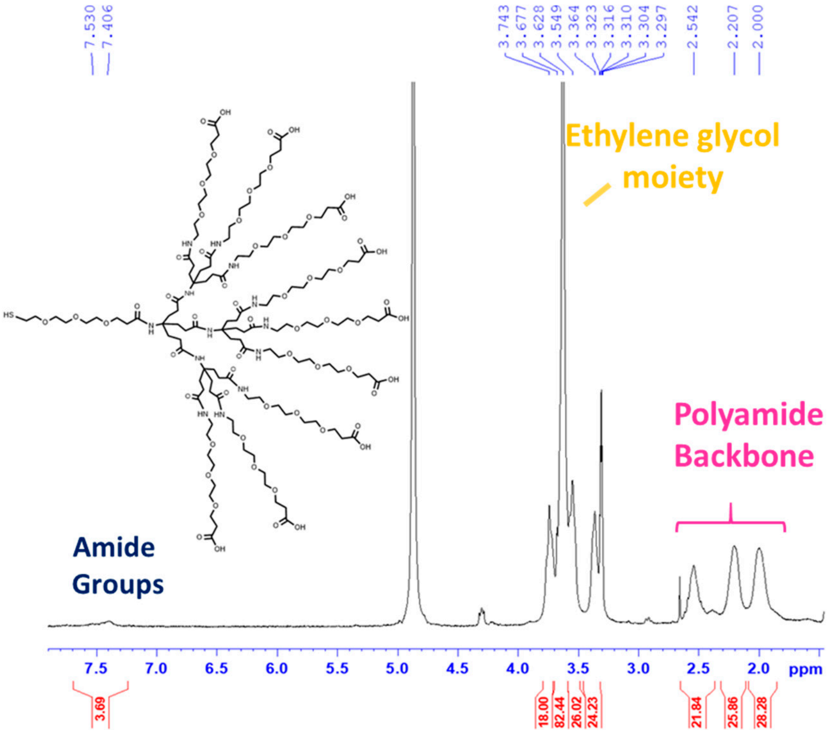

Figure S33. ^{1}H NMR spectrum of compound **13** (600 MHz, D_2O, 25 ºC).

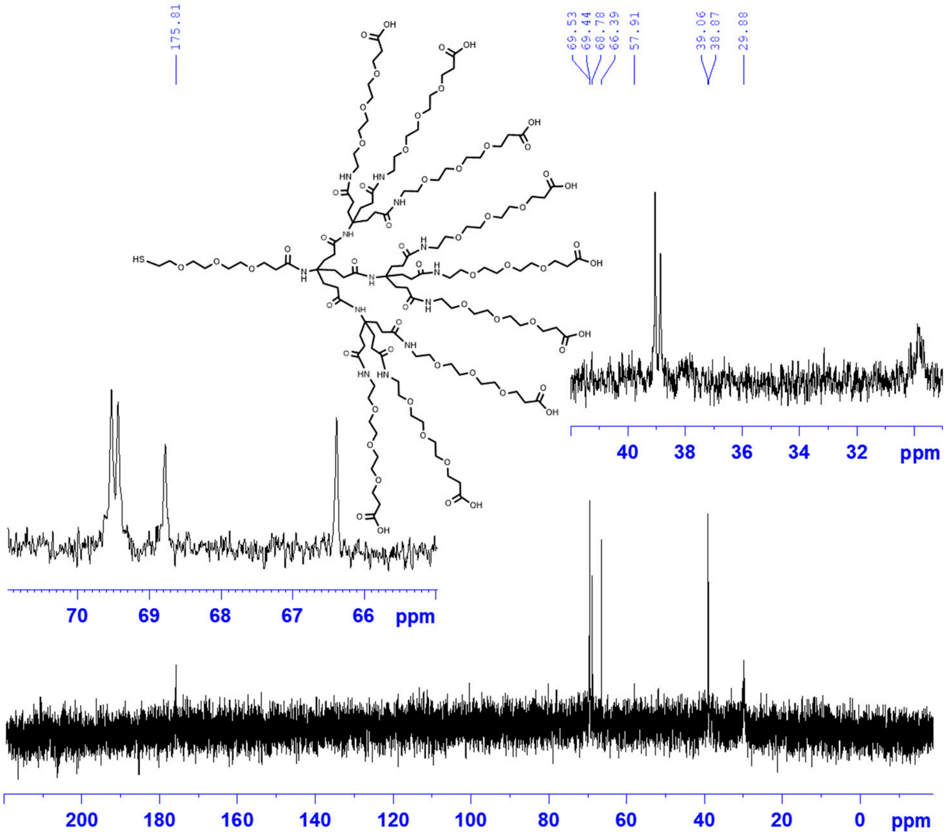

Figure S34. ^{13}C NMR spectrum of compound **13** (600 MHz, D_2O, 25 ºC).

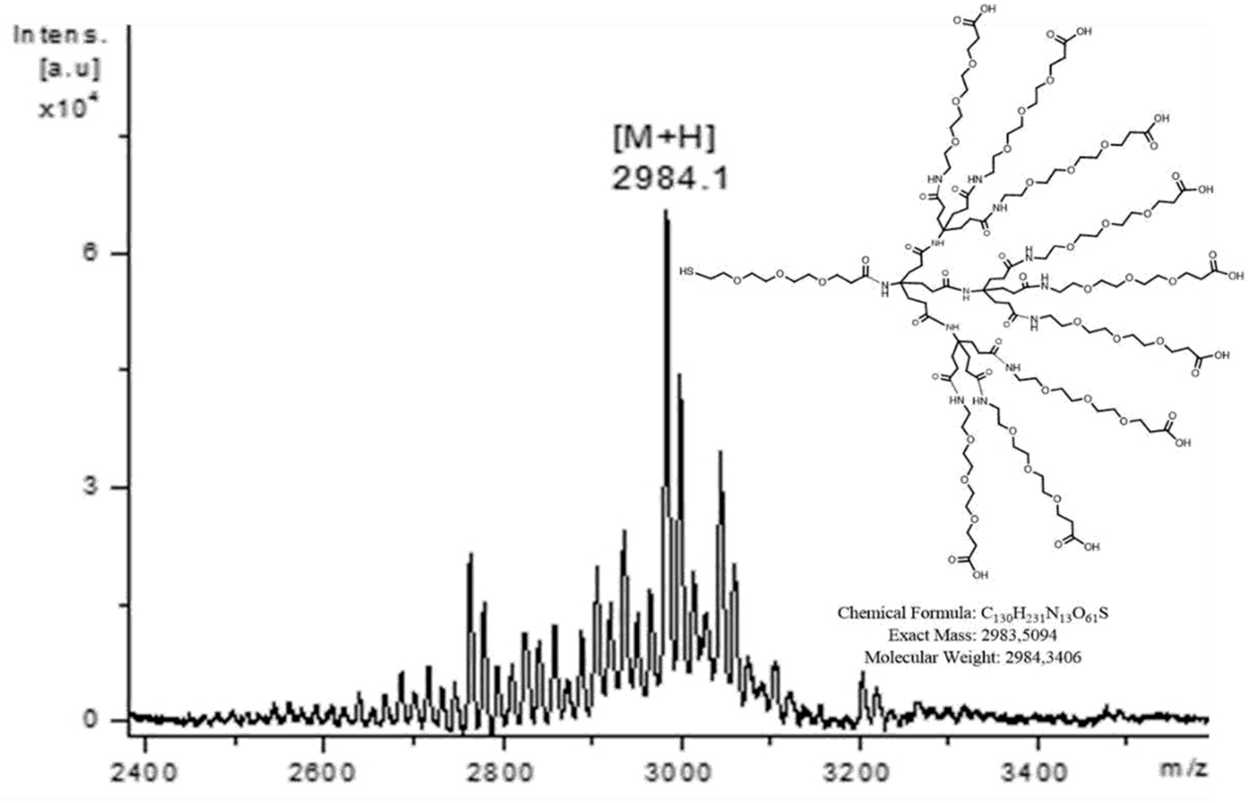

Figure S35. MALDI-TOF mass spectrum of compound **13** (600 MHz, D_2O, 25 ºC).
